# NQQR: scalable qutrit image representation

**DOI:** 10.1038/s41598-026-57304-9

**Published:** 2026-06-23

**Authors:** Mirna Rofail, Rasha Montaser, Ahmed Younes

**Affiliations:** 1 Department of Mathematics and Computer Science, Faculty of Science, Alexandria University, Alexandria, 21526 Egypt; 2 Department of Information Systems, Faculty of Computers and Information Science, Damanhour University, Damanhour, 22511 Egypt; 3 Faculty of Computer Science and Engineering, Alamein International University, New Alamein, 51718 Egypt

**Keywords:** Ternary quantum systems, Ternary quantum image processing, Quantum image representation, Qutrit-based quantum computing, Quantum circuits optimization, Grayscale and RGB quantum images, Mathematics and computing, Optics and photonics, Physics

## Abstract

Ternary quantum image processing (TQIP) integrates qutrit-based quantum computing with image representation, offering a promising pathway for efficient image encoding models. This paper introduces Novel Qutrit Quantum Representation (NQQR) model, a scalable and optimized ternary model for representing grayscale and RGB images of size $3^n \times 3^n$ in a ternary quantum system. The proposed model encodes pixel positions using the superposition of 2*n* qutrits and assigns grayscale or color intensity values using 6 qutrits, while an additional color qutrit and an ancilla qutrit are employed to reduce the complexity of multi-controlled *N*-qutrit gates, thereby lowering the overall quantum cost. Experimental results show that the NQQR achieves a time complexity of $O(n3^{2n})$ and the optimized NQQR model significantly reduces quantum cost, with improvements up to $99.36\%$ for grayscale images and $99.33\%$ for RGB images when compared to equivalent non-optimized qubit/qutrit-based encoding models. In addition, the performance of the proposed model is evaluated under quantum depolarizing noise using MSE and PSNR metrics computed between the original and reconstructed images under different noise levels. These results demonstrate the efficiency and scalability of the proposed NQQR model at the representation level and suggest its potential as a building block for future research in TQIP.

**Supplementary Information:**

The online version contains supplementary material available at 10.1038/s41598-026-57304-9.

## Introduction

Quantum computing expands the boundaries of computational power by leveraging the principles of quantum mechanics such as superposition and entanglement, enabling quantum systems to solve complex problems with a level of efficiency and parallelism that classical computers cannot achieve^1–5^. In addition to traditional binary quantum systems, ternary quantum systems use three-level quantum states (qutrits), which increase data density, reduce circuit complexity, and allow more efficient quantum algorithms ^6–9^. These advantages are particularly important in fields such as image processing^10,11^, cryptography ^12,13^ and machine learning^14,15^, where digital images are fundamental data units requiring efficient storage, manipulation, and retrieval.

Quantum image representation is an essential step in quantum image processing (QIP). While most existing representation models rely on binary qubits^16–21^, ternary quantum systems offer higher scalability and flexibility, providing more compact and efficient encoding of digital images^22–25^. In binary-based quantum systems, the Flexible Representation of Quantum Images (FRQI)^16^, the Novel Enhanced Quantum Representation (NEQR) ^17^, the Multi-Channel Quantum Representation (MCQI)^18^ and the Novel Quantum Representation of Color Digital Images (NCQI)^19^ have been widely used to represent grayscale and RGB images. Despite their effectiveness, binary models require a rapidly increasing number of qubits and more complex circuits as image dimensions grow. To address these scalability challenges, ternary (qutrit-based) models have been proposed as alternative encoding models. The Qutrit Representation of Grayscale Quantum Images (QTRQ) model^22^ introduced the first ternary model for representing grayscale images, followed by circuit-level optimizations that improved the image representation in the QTRQ model^24^. The Ternary Novel Colored Quantum Representation (TNCQR) model^25^ further extended ternary encoding by representing RGB images, lowering time complexity, and reducing quantum cost. However, these ternary models still suffer from several limitations: they are not fully native ternary models, as they are derived from binary models; the quantum cost remains high even after optimization; and as the image dimensions increase ($3^n \times 3^n$), circuit complexity grows rapidly, making practical implementation for real-world images challenging.

Despite the growing interest in QIP, several fundamental challenges remain, including issues related to measurement-induced information loss and unintended entanglement in certain representation models. These challenges limit the practical applicability of many existing approaches. In this work, we focus on improving the efficiency and scalability of ternary quantum image representation, which is a key component in QIP pipelines.

Accordingly, this paper proposes a Novel Quantum Qutrit Representation (NQQR) model. NQQR is a pure ternary quantum representation that enables compact and scalable encoding of $3^n \times 3^n$ grayscale and RGB images using qutrit-based quantum computation. By minimizing multi-qutrit gate usage, and applying optimization techniques, NQQR achieves lower quantum cost and improved scalability compared to relevant ternary and binary models such as QTRQ^22^, TNCQR^25^, FRQI^16^, MCQI^18^, NEQR^17^ and NCQI^19^. The main novelty of the proposed NQQR model lies in its native ternary formulation, which differentiates it from existing qubit-based and binary-derived ternary approaches. By directly exploiting qutrit systems, the model achieves higher information density, improved scalability, and reduced the reliance on complex multi-controlled operations. In addition to efficient image representation, the robustness of quantum image encoding under noise is a critical challenge in practical quantum systems. Therefore, this work also investigates the performance of the proposed model under quantum depolarizing noise using a statistical reconstruction framework based on Monte Carlo sampling. This approach enables effective reduction of quantum noise effects without relying on quantum error correction techniques.

The rest of this paper is organized as follows: Section Preliminaries provides background on quantum ternary logic. Section Related Works reviews existing ternary quantum image representation models. Section The Proposed NQQR Model for Grayscale Images introduces the proposed representation for grayscale images, while Section The Proposed NQQR Model for RGB Images extends the representation to RGB images in ternary quantum system. Section NQQR Optimization Method presents the optimization phases used to reduce the quantum cost of the constructed circuits for both grayscale and RGB images. Section Experimental Results evaluates the performance of the proposed model and compares it with relevant qubit/qutrit-based representation models. Section Performance Evaluation of NQQR Under Quantum Noise presents the performance of the proposed model under different levels of quantum depolarizing noise for both grayscale and RGB images. Finally, Section Conclusion concludes the paper and outlines directions for future work.

## Preliminaries

This section provides a brief background on the fundamental ternary quantum gates that form the basis of the proposed image representation model. These gates operate on ternary quantum system, where each quantum state can exist in 3 distinct levels ($\left| 0\right\rangle$,$\left| 1\right\rangle$, $\left| 2\right\rangle$), offering greater representational capacity compared to binary qubits. This section also presents definitions of the basic computational metrics adopted in this study, namely time complexity and quantum cost in ternary quantum systems.

### Definition 1

The time complexity is the number of sequential layers of gates (non-parallelized operations) that must be executed to realize the circuit (circuit depth)^17,25–28^.

### Definition 2

Quantum cost is a metric that assigns costs to gates according to the ternary quantum system architecture. Quantum cost evaluates complex (*N*-qutrit) gates by decomposing them into elementary ternary gates (one-qutrit and two-qutrit gates) and summing their costs^24,25,28–32^.

### One-qutrit gates

In ternary quantum system there are 7 one-qutrit gates: the 6 ternary shift gates (Z gates)^22–25,29,30,32,33^ and the ternary Hadamard gate (*H*)^22,24,25,34^. Each one-qutrit gate operates on one qutrit to change the qutrit state. The Z gates are: $[+0], [+1], [+2], [01], [02], [12]$. The $[+0]$ gate is the identity gate (*I*). The $[+1]$ gate is a single-shift gate that changes the ternary state by adding 1 mod 3. The $[+2]$ gate is a double-shift gate that changes the ternary state by adding 2 mod 3. The [01] gate is a self-single-shift gate that swaps the ternary state $\left| 0\right\rangle$ and $\left| 1\right\rangle$. The [02] gate is a self-double-shift gate that swaps the ternary state $\left| 0\right\rangle$ and $\left| 2\right\rangle$. The [12] gate is a self-shift gate that swaps the ternary state $\left| 1\right\rangle$ and $\left| 2\right\rangle$^22–25,29,30,32,33^.

The ternary *H* gate is responsible for preparing the ternary superposition states. When applying the *H* gate on a ternary quantum state $\left| \psi \right\rangle =\alpha \left| 0\right\rangle +\beta \left| 1\right\rangle +\gamma \left| 2\right\rangle$ it produces the general form of the ternary superposition states, as shown in Eq (1)^22,24,25,34^,

1 $$\begin{aligned}{ H\left| \psi \right\rangle =\frac{1}{\sqrt{3}}(\alpha +\beta +\gamma )\left| 0\right\rangle +\frac{1}{\sqrt{3}}(\alpha +\beta e^{\frac{2\pi i}{3}}+\gamma e^{\frac{4\pi i}{3}})\left| 1\right\rangle +\frac{1}{\sqrt{3}}(\alpha +\beta e^{\frac{4\pi i}{3}}+\gamma e^{\frac{8\pi i}{3}})\left| 2\right\rangle .} \end{aligned}$$

**Fig. 1:**
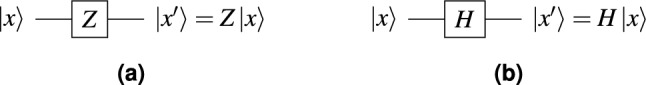
The symbolic notation of one-qutrit gates as a quantum circuit: (**a**) The notation of any *Z* gate. (**b**) The notation of ternary *H* gate.

To maintain reversibility in ternary quantum circuits, each one-qutrit gate must have an inverse gate to reverse its function. The $[+1]$ and $[+2]$ are mutual inverses, which means that they inverse each other’s function. The gates [01], [02], [12], and *H* are self-inverse, which mean that applying the same gate twice results in the identity function ($[+0]$)^24,30,32^. Figure 1 shows the symbolic notation for *Z* and *H* gates as quantum circuits, each with quantum cost equals 1 ($QC=1$)^24,29–32^. Table 1 presents the truth table for the 7 one-qutrit gates: the 6 ternary Z gates and the ternary *H* gate.

**Table 1:** The Truth table for the 7 one-qutrit gates^30,32,34^.

Input	$\mathbf {[+0]}$	$\mathbf {[+1]}$	$\mathbf {[+2]}$	$\mathbf {[01]}$	$\mathbf {[02]}$	$\mathbf {[12]}$	$\mathbf {\textit{H}}$
$\mathbf {\left| 0\right\rangle }$	$\left| 0\right\rangle$	$\left| 1\right\rangle$	$\left| 2\right\rangle$	$\left| 1\right\rangle$	$\left| 2\right\rangle$	$\left| 0\right\rangle$	$\frac{1}{\sqrt{3}}(\left| 0\right\rangle +\left| 1\right\rangle +\left| 2\right\rangle )$
$\mathbf {\left| 1\right\rangle }$	$\left| 1\right\rangle$	$\left| 2\right\rangle$	$\left| 0\right\rangle$	$\left| 0\right\rangle$	$\left| 1\right\rangle$	$\left| 2\right\rangle$	$\frac{1}{\sqrt{3}}(\left| 0\right\rangle +e^{\frac{2\pi i}{3}}\left| 1\right\rangle +e^{\frac{4\pi i}{3}}\left| 2\right\rangle )$
$\mathbf {\left| 2\right\rangle }$	$\left| 2\right\rangle$	$\left| 0\right\rangle$	$\left| 1\right\rangle$	$\left| 2\right\rangle$	$\left| 0\right\rangle$	$\left| 1\right\rangle$	$\frac{1}{\sqrt{3}}(\left| 0\right\rangle +e^{\frac{4\pi i}{3}}\left| 1\right\rangle +e^{\frac{8\pi i}{3}}\left| 2\right\rangle )$

### Two-qutrit M-S gates

**Fig. 2:**
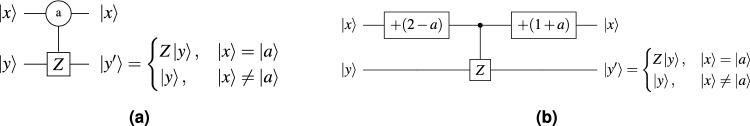
Symbolic notations for the two-qutrit gates as a quantum circuit: (**a**) The notation of generalized two-qutrit gate when the control qutrit $\left| x\right\rangle$ is in state $\left| a\right\rangle \in \{\left| 0\right\rangle ,\left| 1\right\rangle ,\left| 2\right\rangle \}$. (**c**) The decomposition of generalized two-qutrit gate.

The two-qutrit gates proposed by Muthukrishnan and Stroud^35^ operate on 2 qutrits: a control qutrit and a target qutrit. The *Z* gate on the target qutrit is applied when the control qutrit satisfies the conditional state $\left| a\right\rangle$, where $a \in \{0,1,2\}$ as shown in Figure 2a. The *QC* for two-qutrit gates is $2k+1$, where *k* is the number of controls not in state $\left| 2\right\rangle$^22,24,25,29–33,36^.

Figure 2b shows the decomposition of generalized two-qutrit gate when $\left| a\right\rangle \ne \left| 2\right\rangle$ into 3 gates: 2 one-qutrit gates and 1 two-qutrit gate with control qutrit in state $\left| 2\right\rangle$. In this figure, $[+(2-a)]$ and $[+(1+a])$ represent the gates $[+((2-a)\,mod\,3)]$ and $[+((1+a)\,mod\,3)]$, respectively, where $a\in \{\left| 0\right\rangle ,\left| 1\right\rangle ,\left| 2\right\rangle \}$.

### Generalized N-qutrit M-S gate

**Fig. 3:**
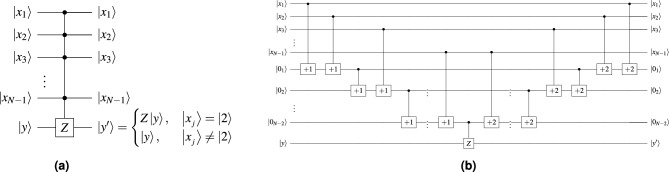
Symbolic notation for the *N*-qutrit gate as a quantum circuit: (**a**) The default notation of *N*-qutrit gate with $N - 1$ control qutrits $\left| x_{_j}\right\rangle$ all in state $\left| 2\right\rangle$, where $j \in \{1,2,3,...,N-1\}$ (**b**) The decomposition of *N*-qutrit gate.

The generalized *N*-qutrit M-S gate operates on *N* qutrits. The *Z* gate on the target qutrit is activated when all $N-1$ control qutrits are in state $\left| 2\right\rangle$, as shown in Figure 3a, with $QC=4N-7$^22,25^, where $N \ge 2$ qutrits.

To decompose the *N*-qutrit gate, add $N-2$ ancilla qutrits in state $\left| 0\right\rangle$, then the $N-1$ control qutrits are decomposed into $2N-4$ two-qutrit gates with a target $[+1]$ gate, followed by 1 two-qutrit gate with a target *Z* gate, and finally $2N-4$ two-qutrit gates with a target $[+2]$ gate. Figure 3b illustrates the decomposition of the *N*-qutrit M-S gate^22,29,31^.

**Fig. 4:**
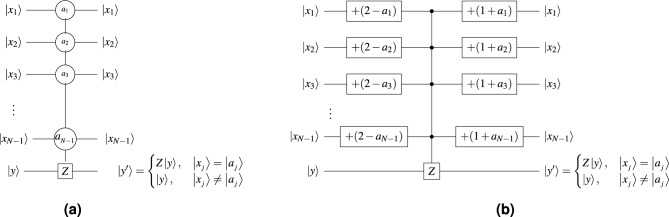
Symbolic notation for the generalized *N*-qutrit gate as a quantum circuit: (**a**) The generalized *N*-qutrit gate with control qutrits $\left| x_{_j}\right\rangle$ function according to state $\left| a_{_j}\right\rangle \in \{\left| 0\right\rangle , \left| 1\right\rangle ,\left| 2\right\rangle \}$, where $j \in \{1, 2,3,...,N-1\}$ (**b**) The decomposition of generalized *N*-qutrit gate.

While qutrit controls can operate in any state: $\left| 0\right\rangle$, $\left| 1\right\rangle$, or $\left| 2\right\rangle$, an *N*-qutrit gate can operate with any control state, as shown in Fig. 4a. Figure 4b illustrates the decomposition of a generalized *N*-qutrit gate. The QC for this gate is calculated by $QC = 4N + 2k - 7$^22,25^, where *N* is the number of qutrits used in the gate, and *k* is the number of controls not in state $\left| 2\right\rangle$.

## Related works

This section presents recent relevant models for representing digital images in ternary quantum system such as: the QTRQ^22^ and TNCQR models^25^.

### QTRQ representation model

**Fig. 5:**
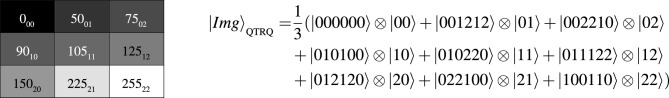
A $3\times 3$ grayscale image with its corresponding quantum image state $\left| Img\right\rangle _{_\text {QTRQ}}$ represented using QTRQ model.

The QTRQ model^22^ is designed to encode grayscale images into a ternary quantum system. It employs $2n + q$ qutrits to represent a $3^n \times 3^n$ grayscale image, where the grayscale value in range $[0-255]$ is encoded using $q = 6$ qutrits per pixel, instead of $q = 8$ qubits used in binary-based models. The mathematical expression for representing a $3^n \times 3^n$ grayscale quantum image using the QTRQ model is expressed as follows,

2 $$\begin{aligned} \left| Img\right\rangle _{_\text {QTRQ}}=\dfrac{1}{3^n}\sum _{Y=0}^{3^n-1}\sum _{X=0}^{3^n-1}\left| f(Y,X)\right\rangle \left| YX\right\rangle =\dfrac{1}{3^n}\sum _{Y=0}^{3^n-1}\sum _{X=0}^{3^n-1}\bigotimes _{l=0}^{q-1}\left| C^{^{\,l}}_{_{YX}}\right\rangle \left| YX\right\rangle , \end{aligned}$$

where $\left| f(Y,X)\right\rangle$ denotes the grayscale value of the pixel located at position (*Y*, *X*), encoded as a ternary sequence $C^{0}_{YX} C^{1}_{YX} \ldots C^{q-1}_{YX}$, where each $\left| C^{^{\,l}}_{_{YX}}\right\rangle \in {\left| 0\right\rangle , \left| 1\right\rangle , \left| 2\right\rangle }$. Figure 5 presents a $3 \times 3$ grayscale image and its corresponding quantum image state $\left| Img\right\rangle _{_\text {QTRQ}}$, where each pixel is indexed by its (*Y*, *X*) position and assigned a grayscale value in the range $[0-255]$. The quantum circuit constructed using the QTRQ model records a total quantum cost of $QC = 203$^22^. Additional optimization techniques described in ^22^ further reduce the quantum cost to $QC = 107$.

**Fig. 6:**
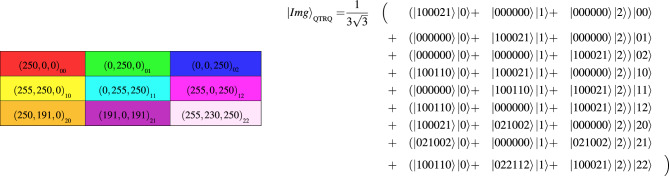
A $3\times 3$ RGB image with its corresponding quantum image state $\left| Img\right\rangle _{_\text {QTRQ}}$ represented using QTRQ model^25^.

The QTRQ model can be extended to represent $3^n \times 3^n$ RGB digital images by adding one additional qutrit to encode the 3 color channels^25^. In this extension, the Red, Green, and Blue channels are represented by the 3 qutrit states ($\left| 0\right\rangle$, $\left| 1\right\rangle$, and $\left| 2\right\rangle$) through the application of the ternary Hadamard (*H*) gate on the color qutrit. Therefore, the extended model requires $2n + q + 1$ qutrits to store a $3^n \times 3^n$ RGB image with a color range of $[0-255]$, where $q = 6$ qutrits are used to represent the color value of each channel per pixel. Figure6 presents a $3\times 3$ RGB image and the corresponding quantum image state $\left| Img\right\rangle _{_\text {QTRQ}}$ for the RGB image. The time complexity for preparing a quantum image using the QTRQ model is $O(qn3^{2n})$.

Although the QTRQ model provides a compact way to represent quantum images using qutrits instead of qubits, it has several limitations. First, the model is not a true ternary representation but a conversion from the NEQR binary model. Second, it uses a limited set of generalized N-qutrit gates with 2*n* or $2n+1$ controls, which increases the circuit depth and results in a high time complexity of $O(q n 3^{2n})$. Third, the quantum cost is still high even for small images. Finally, as the image size grows to $3^n \times 3^n$, the number of qutrits and the circuit complexity increase quickly, making it difficult to scale to larger or real-world images.

### TNCQR representation model

The TNCQR model is designed to encode $3^n \times 3^n$ RGB digital images with time complexity $O(n3^{2n})$ and a lower quantum cost compared to the QTRQ model^25^. In the TNCQR model, 7 qutrits are used to encode the color intensity of each pixel. Therefore, the model requires $2n + q + 2$ qutrits to store a $3^n \times 3^n$ RGB image, where $q = 6$ qutrits are used to represent the color range $[0-255]$ for each pixel. An ancilla qutrit is used to load the position of each pixel and assign its value within the range $[0-255]$ using up to 5 two-qutrit gates. The inclusion of this ancilla qutrit reduces the overall circuit cost by reducing the number of complex *N*-qutrit gates required, where $N \ge 3$. The representative expression for storing a $3^n \times 3^n$ RGB quantum image is given in Eq (3), and the corresponding quantum image state $\left| Img\right\rangle _{_\text {TNCQR}}$ for the $3 \times 3$ RGB image in Figure 6 is shown in Eq (4). The constructed quantum circuit for this image using the TNCQR model has $QC = 267$. An optimization algorithm has been applied to reduce the quantum cost to $QC = 170$^25^.

3 $$\begin{aligned} \begin{aligned} \left| Img\right\rangle _{_\text {TNCQR}}&=\dfrac{1}{3^n\sqrt{3}}\sum _{Y=0}^{3^n-1}\sum _{X=0}^{3^n-1}\left| f(Y,X)\right\rangle \left| 0\right\rangle _{\text {aux}}\left| YX\right\rangle \\&=\dfrac{1}{3^n\sqrt{3}}\sum _{Y=0}^{3^n-1}\sum _{X=0}^{3^n-1}\Big (\left| R_{YX}\right\rangle \left| 0\right\rangle +\left| G_{YX}\right\rangle \left| 1\right\rangle +\left| B_{YX}\right\rangle \left| 2\right\rangle \Big )\left| 0\right\rangle _{\text {aux}}\left| YX\right\rangle . \end{aligned} \end{aligned}$$

4 $$\begin{aligned} \begin{aligned} \left| Img\right\rangle _{_\text {TNCQR}}=&\frac{1}{3\sqrt{3}}&\Bigl (  (\left| 100021\right\rangle \left| 0\right\rangle+ & \left| 000000\right\rangle \left| 1\right\rangle+ & \left| 000000\right\rangle \left| 2\right\rangle )&\quad \left| 0\right\rangle _{aux}\left| 00\right\rangle \\ & + & (\left| 000000\right\rangle \left| 0\right\rangle+ & \left| 100021\right\rangle \left| 1\right\rangle+ & \left| 000000\right\rangle \left| 2\right\rangle )&\quad \left| 0\right\rangle _{aux}\left| 01\right\rangle \\ & + & (\left| 000000\right\rangle \left| 0\right\rangle+ & \left| 000000\right\rangle \left| 1\right\rangle+ & \left| 100021\right\rangle \left| 2\right\rangle )&\quad \left| 0\right\rangle _{aux}\left| 02\right\rangle \\ & + & (\left| 100110\right\rangle \left| 0\right\rangle+ & \left| 100021\right\rangle \left| 1\right\rangle+ & \left| 000000\right\rangle \left| 2\right\rangle )&\quad \left| 0\right\rangle _{aux}\left| 10\right\rangle \\ & + & (\left| 000000\right\rangle \left| 0\right\rangle+ & \left| 100110\right\rangle \left| 1\right\rangle+ & \left| 100021\right\rangle \left| 2\right\rangle )&\quad \left| 0\right\rangle _{aux}\left| 11\right\rangle \\ & + & (\left| 100110\right\rangle \left| 0\right\rangle+ & \left| 000000\right\rangle \left| 1\right\rangle+ & \left| 100021\right\rangle \left| 2\right\rangle )&\quad \left| 0\right\rangle _{aux}\left| 12\right\rangle \\ & + & (\left| 100021\right\rangle \left| 0\right\rangle+ & \left| 021002\right\rangle \left| 1\right\rangle+ & \left| 000000\right\rangle \left| 2\right\rangle )&\quad \left| 0\right\rangle _{aux}\left| 20\right\rangle \\ & + & (\left| 021002\right\rangle \left| 0\right\rangle+ & \left| 000000\right\rangle \left| 1\right\rangle+ & \left| 021002\right\rangle \left| 2\right\rangle )&\quad \left| 0\right\rangle _{aux}\left| 21\right\rangle \\ & + & (\left| 100110\right\rangle \left| 0\right\rangle+ & \left| 022112\right\rangle \left| 1\right\rangle+ & \left| 100021\right\rangle \left| 2\right\rangle )&\quad \left| 0\right\rangle _{aux}\left| 22\right\rangle\Bigr ) \end{aligned} \end{aligned}$$

The TNCQR model is also applicable to grayscale images. Implementing the $3 \times 3$ grayscale image shown in Figure 5, results in a quantum circuit with initial quantum cost $QC = 149$, which is reduced to $QC = 85$ using the optimization algorithm, matching the quantum cost reported in^24^.

Although the TNCQR model is better than the QTRQ model in terms of quantum cost and time complexity, it still has some limitations. First, the model is not fully native ternary representation but is derived from the ENEQR binary model. Second, the quantum cost remains relatively high even after optimization. To overcome these limitations, this paper proposes the NQQR model, a novel ternary representation that exploits qutrit-based computation to provide a more compact and resource-efficient encoding of $3^n \times 3^n$ grayscale and RGB images in quantum systems.

## The proposed NQQR model for grayscale images

This section introduces the proposed Novel Qutrit Quantum Representation model (NQQR) for representing grayscale images in ternary quantum system. To validate the proposed representation at the circuit level, all quantum circuits are implemented and evaluated using Google Cirq ^37^ for ternary quantum systems.

### NQQR image representation model

**Fig. 7:**
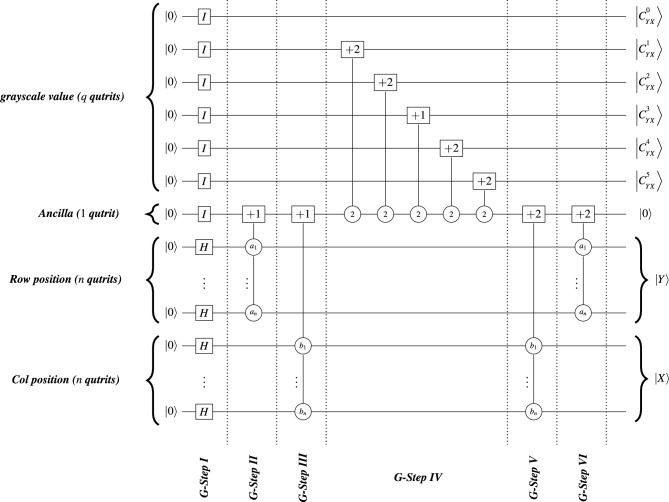
The general ternary quantum circuit for representing a $3^n\times 3^n$ grayscale image using the NQQR model.

The NQQR model represents a $3^n \times 3^n$ grayscale image in ternary quantum system, as shown in the general ternary quantum circuit in Figure 7. The representation process consists of the following G-Steps, where each ”G-Step” refers to a specific stage in encoding the grayscale image using the proposed NQQR model.

- **G-Step I**: The $2n+q+1$ qutrits are initialized to $\left| 0\right\rangle$, then $H^{\otimes {2n}}$ gates are applied on the 2*n* position qutrits.
- **G-Step II**: A generalized *N*-qutrit gate with *n* control qutrits (row position *Y*) and a $[+1]$ target gate is used to update the ancilla qutrit from $\left| 0\right\rangle$ to $\left| 1\right\rangle$ (Enable row *Y*).
- **G-Step III**: Another generalized *N*-qutrit gate with *n* control qutrits (column position *X*) and a $[+1]$ target gate is used to update the ancilla qutrit from $\left| 1\right\rangle$ to $\left| 2\right\rangle$ (Enable column *X*).
- **G-Step IV**: The grayscale value of the pixel (*Y*, *X*), in range $[0-255]$, is assigned using up to 5 two-qutrit gates, where each gate uses the ancilla qutrit in state $\left| 2\right\rangle$ as the control qutrit and applies a $[+1]$ or $[+2]$ target gate to one of the *q* qutrits.
- **G-Step V**: A generalized *N*-qutrit gate with *n* control qutrits (column position *X*) and a $[+2]$ target gate is used to reverse the effect of G-Step III, returning the ancilla state from $\left| 2\right\rangle$ to $\left| 1\right\rangle$, preparing the representation of the next pixel in the same row *Y* (Disable column *X*).
- *Repeat G-Steps III−V  to assign the grayscale value for all pixels in the same row*$\textit{Y}$.
- **G-Step VI**: A generalized *N*-qutrit gate with *n* control qutrits (row position *Y*) and a $[+2]$ target gate is used to reverse the effect of Step II, returning the ancilla state from $\left| 1\right\rangle$ to $\left| 0\right\rangle$, preparing the representation of the next row of pixels in the image (Disable row *Y*).
- *The G-Steps II−VI  are repeated to represent all pixels in the image.*

The detailed explanation for representing a $3^n\times 3^n$ gray image using the NQQR is presented. **G-Step I** initializes the grayscale image as the quantum state $\left| \Psi _0\right\rangle$ with $q+1+2n$ qutrits all initialized to $\left| 0\right\rangle$, as shown in Eq (5). The 6 qutrits ($q=6$) are used to store the grayscale value for each pixel (since $3^6=729$ covers the range from 0 to 255), 2*n* qutrits are used to encode the pixel positions and 1 ancilla qutrit.

5 $$\begin{aligned} \begin{aligned} \left| \Psi _0\right\rangle = \underbrace{\left| 000000\right\rangle }_{q\text { qutrits } } \otimes \underbrace{\left| 0\right\rangle }_{\text { ancilla qutrit }} \otimes \underbrace{\left| 0\right\rangle ^{\otimes 2n}}_{2n\text { position qutrits }}. \end{aligned} \end{aligned}$$

The $U_1$ operator in Eq(6) is then applied on $\left| \Psi _0\right\rangle$ state to encode the position information for $3^{2n}$ pixels. This operator applies $H^{\otimes 2n}$ gates on the 2*n* position qutrits, creating a superposition of all $3^{2n}$ pixel positions. The operator transforms the initial state $\left| \Psi _0\right\rangle$ into the intermediate state $\left| \Psi _1\right\rangle$ as shown in Eq (7), where $\left| YX\right\rangle$ represents a pixel at position (*Y*, *X*) as row *Y*, column *X*.

6 $$\begin{aligned} \begin{aligned} U_1 = I^{\otimes q+1} \otimes H^{\otimes 2n}. \end{aligned} \end{aligned}$$

7 $$\begin{aligned} \begin{aligned} \left| \Psi _1\right\rangle&= U_1 \left| \Psi _0\right\rangle \\&= (I\left| 0\right\rangle )^{\otimes q+1} \otimes (H\left| 0\right\rangle )^{\otimes 2n}\\&= \frac{1}{3^n} \sum ^{3^n-1}_{Y=0} \sum ^{3^n-1}_{X=0} \quad \underbrace{\left| 000000\right\rangle }_{q \textit{ qutrits }} \quad \otimes \underbrace{\left| 0\right\rangle }_{\textit{ancilla qutrit}} \otimes \quad \underbrace{\left| YX\right\rangle }_{\textit{position information}}. \end{aligned} \end{aligned}$$

The grayscale value for each pixel is assigned using $U_{_{YX}}$ operator (**G-Steps II, III, IV**), as shown in Eq (8). This operator operates iteratively on each row of pixels in the image, starting from Row$_{_0}$ till Row$_{_{3^n-1}}$. For each Row$_{_Y}$, the gray-setting quantum operation $\varpi _{_{YX}}$ assigns the grayscale value for all pixels in that row, where $\left| YX\right\rangle$ represents the pixel currently being processed and $\left| vu\right\rangle$ represents the other pixels in the image that are not currently being updated. The $\varpi _{_{Y X}}$ operation consists of several sub-operations $\Re _{_{Y}}, \varsigma _{_{X}}, \Omega _{_{Y X}}$ as expressed in Eq (9),

8 $$\begin{aligned} U_{_{Y X}} = \Bigl (I \otimes \sum ^{3^n-1}_{v=0} \sum ^{3^n-1}_{\begin{array}{c} u=0\\ vu \ne Y X \end{array}} \left| vu\right\rangle \left\langle vu\right| \Bigr ) + \varpi _{_{Y X}} \otimes \left| Y X\right\rangle \left\langle Y X\right| , \end{aligned}$$

9 $$\begin{aligned} \varpi _{_{Y X}} = \, \Re _{_{Y}}\Bigl (\varsigma _{_{X}} \Bigl (\Omega _{_{Y X}}\Bigr ) \, \varsigma _{_{X}} \Bigr )\Re _{_{Y}}. \end{aligned}$$

The sub-operation $\Re _{_{Y}}$ is an *N*-qutrit gate that uses the row position *Y* of the pixel (*Y*, *X*) as *n* control qutrits to update the state of the ancilla qutrit from $\left| 0\right\rangle$ to $\left| 1\right\rangle$ (**G-Step II**). The sub-operation $\varsigma _{_{X}}$ is an *N*-qutrit gate that uses the column position *X* of the pixel (*Y*, *X*) as *n* control qutrits to update the state of the ancilla qutrit from $\left| 1\right\rangle$ to $\left| 2\right\rangle$ (**G-Step III**). The sub-operation $\Omega _{_{Y X}}$ assigns the grayscale value in ternary representation over the $q=6$ qutrits to the pixel (*Y*, *X*) in range $[0 - 255]$ (**G-Step IV**). Therefore, the sub-operation $\Omega _{_{Y X}}$ consists of *q* unitary operations as follows,

10 $$\begin{aligned} \Omega _{_{Y X}}=\bigotimes ^{^{q-1}}_{_{l=0}}\Omega _{_{Y X}}^{^{l}}, \end{aligned}$$

where the $\Omega _{_{Y X}}^{^{l}}$ unitary operator is a 1 two-qutrit M-S gate that is applied to 1 of the *q* qutrits to change its state from $\left| 0\right\rangle$ to $\left| 1\right\rangle$ or $\left| 2\right\rangle$ according to the pixel’s grayscale value as follows,

11 $$\begin{aligned} \Omega _{_{Y X}}^{^{l}}:\left| 0\right\rangle \rightarrow \left| 0\oplus C^{^{^l}}_{_{Y X}}\right\rangle . \end{aligned}$$

Applying $\Omega _{_{Y X}}$ on the *q* qutrits to set the grayscale value for the pixel (*Y*, *X*) is expressed as follows,

12 $$\begin{aligned} \begin{aligned} \Omega _{_{Y X}} (\left| 0\right\rangle ^{\otimes q})&= \Biggl (\bigotimes ^{^{q-1}}_{_{l=0}}\Omega _{_{Y X}}^{^{l}}\Biggr ) (\left| 0\right\rangle ^{\otimes q})\\&= \Big (\Omega _{_{Y X}}^{^0}\otimes \Omega _{_{Y X}}^{^1}\otimes ...\otimes \Omega _{_{Y X}}^{^{q-1}}\Big )(\left| 0\right\rangle \left| 0\right\rangle ...\left| 0\right\rangle )\\&= \left| 0\oplus C_{_{Y X}}^{^0}\right\rangle \otimes \left| 0\oplus C_{_{Y X}}^{^1}\right\rangle \otimes ... \otimes \left| 0\oplus C_{_{Y X}}^{^{q-1}}\right\rangle \\&= \left| C_{_{Y X}}^{^0} C_{_{Y X}}^{^1} \, ... \, C_{_{Y X}}^{^{q-1}}\right\rangle \\&=\left| f(Y,X)\right\rangle , \end{aligned} \end{aligned}$$

where the state $\left| f(Y,X)\right\rangle$ represents the assigned grayscale value in ternary representation over the *q* qutrits for the pixel (*Y*, *X*). After assigning the grayscale value using $\Omega _{_{Y X}}$, another $\varsigma _{_{X}}$ gate is used to update the state of the ancilla qutrit from $\left| 2\right\rangle$ to $\left| 1\right\rangle$ to prepare the representation of the next pixel in the same row (**G-Step V**). Finally, the ancilla qutrit state is returned to $\left| 0\right\rangle$ by applying the $\Re _{_{Y}}$ gate, preparing it for the representation of the next row of pixels in the image (**G-Step VI**).

Note that the sub-operations $\Re _{Y}$ and $\varsigma _{X}$ are applied iteratively on all pixels in a $3^n \times 3^n$ image, with $\Re _{Y}$ applied row by row from Row$_{_0}$ to Row$_{_{3^n-1}}$ and $\varsigma _{X}$ applied column by column within each row from Col$_{_0}$ to Col$_{_{3^n-1}}$.

Each of the $\varpi _{_{YX}}$ sub-operations is implemented in the ternary quantum circuit for the NQQR model as follows: the sub-operations $\Re _{_{Y}}$ and $\varsigma _{_{X}}$ are generalized *N*-qutrit M-S gates, each with *n* control and 1 target qutrits, where *n* determines the size of $3^n\times 3^n$ image. The sub-operation $\Omega _{_{Y X}}$ assigns the grayscale value for 1 pixel over the *q* qutrits and is implemented using up to 5 two-qutrit M-S gates, each with a control qutrit set to $\left| 2\right\rangle$, since representing values from 0 to 255 in ternary requires modifying up to 5 ternary digits. Applying the $U_{YX}$ operator on the quantum state $\left| \Psi _1\right\rangle$ updates the grayscale value of the specified pixel (*Y*, *X*), resulting in the transformed state $\left| \Psi _2\right\rangle$, as shown in Eq (13),

13 $$\begin{aligned} \begin{aligned} \left| \Psi _2\right\rangle&=\,U_{_{YX}} (\left| \Psi _1\right\rangle )\\&=U_{_{YX}}\Biggl (\frac{1}{3^n} \sum ^{3^n-1}_{v=0} \sum ^{3^n-1}_{u=0} \left| 0\right\rangle ^{\otimes q+1} \otimes \left| vu\right\rangle \Biggr )\\&=\frac{1}{3^n}\, U_{_{YX}} \Biggl (\sum ^{3^n-1}_{v=0} \sum ^{3^n-1}_{\begin{array}{c} u=0\\ \\ vu \ne Y X \end{array}} \left| 0\right\rangle ^{\otimes q+1} \otimes \left| vu\right\rangle + \left| 0\right\rangle ^{\otimes q+1} \otimes \left| YX\right\rangle \Biggr )\\&=\frac{1}{3^n} \Biggl (\sum ^{3^n-1}_{v=0} \sum ^{3^n-1}_{\begin{array}{c} u=0\\ \\ vu \ne Y X \end{array}} \left| 0\right\rangle ^{\otimes q+1} \otimes \left| vu\right\rangle + \varpi _{_{YX}} \otimes \left| 0\right\rangle ^{\otimes q+1} \otimes \left| YX\right\rangle \Biggr )\\&=\frac{1}{3^n} \Biggl (\sum ^{3^n-1}_{v=0} \sum ^{3^n-1}_{\begin{array}{c} u=0\\ \\ vu \ne Y X \end{array}} \left| 0\right\rangle ^{\otimes q+1} \otimes \left| vu\right\rangle + \Re _{_{Y}}\Bigl (\varsigma _{_{X}} \Bigl (\bigotimes ^{q-1}_{l=0}\Omega ^{^l}_{_{YX}}\Bigr ) \varsigma _{_{X}}\Bigr )\Re _{_{Y}} \otimes \left| 0\right\rangle ^{\otimes q+1} \otimes \left| YX\right\rangle \Biggr )\\&=\frac{1}{3^n} \Biggl (\sum ^{3^n-1}_{v=0} \sum ^{3^n-1}_{\begin{array}{c} u=0\\ \\ vu \ne Y X \end{array}} \left| 0\right\rangle ^{\otimes q+1} \otimes \left| vu\right\rangle + \Re _{_{Y}}\Bigl (\varsigma _{_{X}} \Bigl (\left| C_{_{Y X}}^{^0} C_{_{Y X}}^{^1} \, ... \, C_{_{Y X}}^{^{q-1}}\right\rangle \Bigr ) \varsigma _{_{X}}\Bigr )\Re _{_{Y}} \otimes \left| 0\right\rangle _{\textit{aux}} \otimes \left| YX\right\rangle \Biggr )\\&=\frac{1}{3^n}\Biggl (\sum ^{3^n-1}_{v=0} \sum ^{3^n-1}_{\begin{array}{c} u=0\\ \\ vu \ne Y X \end{array}} \left| 0\right\rangle ^{\otimes q+1}\left| vu\right\rangle +\left| f(Y,X)\right\rangle \left| 0\right\rangle _{aux}\left| YX\right\rangle \Biggr ).\\ \end{aligned} \end{aligned}$$

The $U_{_{YX}}$ operator assigns the grayscale value to each pixel in a single row of the image, with each pixel updated at its corresponding position. Therefore, $U_2$ is the quantum operator that assigns the grayscale values for all $3^{2n}$ image pixels. The $U_2$ operator consists of $3^{n}$ row-wise sub-operations to assign the grayscale values for all pixels row by row for the whole image as follows,

14 $$\begin{aligned} U_2=\prod _{0}^{3^n-1} U_{_{YX}}. \end{aligned}$$

So, applying $U_2$ on the state $\left| \Psi _1\right\rangle$ gives the final state $\left| \Psi _3\right\rangle$, which is the quantum gray image of the NQQR model as shown in Eq (15). The state $\left| \Psi _2\right\rangle$ represents the application of the $U_{_{YX}}$ operator on 1 pixel to assign its value, while $\left| \Psi _3\right\rangle$ represents the application of the $U_2$ operator which assigns the grayscale values to all $3^{2n}$ pixels.

15 $$\begin{aligned} \begin{aligned} \left| \Psi _3\right\rangle&=U_2\left| \Psi _1\right\rangle \\&=\frac{1}{3^n}\sum _{Y=0}^{3^n-1}\sum _{X=0}^{3^n-1} \varpi _{_{YX}} \otimes \left| 0\right\rangle ^{\otimes q+1} \otimes \left| YX\right\rangle \\&=\frac{1}{3^n}\sum _{Y=0}^{3^n-1} \Re _{_Y} \Big ( \sum _{X=0}^{3^n-1} \varsigma _{_X} \Bigl (\sum ^{q-1}_{l=0}\Omega ^{^l}_{_{YX}} \otimes \left| 0\right\rangle ^{\otimes q+1} \otimes \left| YX\right\rangle \Bigr ) \varsigma _{_X} \Big ) \Re _{_Y} \\&=\frac{1}{3^n}\sum _{Y=0}^{3^n-1} \Re _{_Y} \Big ( \sum _{X=0}^{3^n-1} \varsigma _{_X} \Bigl (\left| C_{_{Y X}}^{^0} C_{_{Y X}}^{^1} \, ... \, C_{_{Y X}}^{^{q-1}}\right\rangle \otimes \left| 0\right\rangle _{\textit{aux}} \otimes \left| YX\right\rangle \Bigr ) \varsigma _{_X} \Big ) \Re _{_Y} \\&=\frac{1}{3^n}\sum _{Y=0}^{3^n-1}\sum _{X=0}^{3^n-1} \left| f(Y,X)\right\rangle \left| 0\right\rangle _{\textit{aux}}\left| YX\right\rangle . \end{aligned} \end{aligned}$$

The state $\left| \Psi _3\right\rangle$, which represents a $3^n\times 3^n$ grayscale image, is expressed in detail in Eq (16). This equation illustrates that the NQQR model selects a row of pixels *Y*, and within this row, iterates over each column $X_\ell$ to identify the pixel at position $(Y,X_\ell )$ and assign its corresponding grayscale value, as follows,

16 $$\begin{aligned} \begin{aligned} \left| \Psi _3\right\rangle =&\Bigl (\Re _{_0}\Bigr )\Bigl (\varsigma _{_0}\Bigr )\Bigl (\bigotimes ^{q-1}_{l=0}\Omega ^{^l}_{_{00}}\Bigr ) \Bigl (\varsigma _{_0}\Bigr )\Bigl (\varsigma _{_1}\Bigr )\Bigl (\bigotimes ^{q-1}_{l=0}\Omega ^{^l}_{_{01}}\Bigr ) \Bigl (\varsigma _{_1}\Bigr ) \cdots \Bigl (\varsigma _{_\ell }\Bigr )\Bigl (\bigotimes ^{q-1}_{l=0}\Omega ^{^l}_{_{0\ell }}\Bigr ) \Bigl (\varsigma _{_\ell }\Bigr ) \Bigl (\Re _{_0}\Bigr )+\\&\Bigl (\Re _{_1}\Bigr )\Bigl (\varsigma _{_0}\Bigr )\Bigl (\bigotimes ^{q-1}_{l=0}\Omega ^{^l}_{_{10}}\Bigr ) \Bigl (\varsigma _{_0}\Bigr )\Bigl (\varsigma _{_1}\Bigr )\Bigl (\bigotimes ^{q-1}_{l=0}\Omega ^{^l}_{_{11}}\Bigr ) \Bigl (\varsigma _{_1}\Bigr ) \cdots \Bigl (\varsigma _{_\ell }\Bigr )\Bigl (\bigotimes ^{q-1}_{l=0}\Omega ^{^l}_{_{1\ell }}\Bigr ) \Bigl (\varsigma _{_\ell }\Bigr ) \Bigl (\Re _{_1}\Bigr )+\\&\vdots \\&\Bigl (\Re _{_\ell }\Bigr )\Bigl (\varsigma _{_0}\Bigr )\Bigl (\bigotimes ^{q-1}_{l=0}\Omega ^{^l}_{_{\ell 0}}\Bigr ) \Bigl (\varsigma _{_0}\Bigr )\Bigl (\varsigma _{_1}\Bigr )\Bigl (\bigotimes ^{q-1}_{l=0}\Omega ^{^l}_{_{\ell 1}}\Bigr ) \Bigl (\varsigma _{_1}\Bigr ) \cdots \Bigl (\varsigma _{_\ell }\Bigr )\Bigl (\bigotimes ^{q-1}_{l=0}\Omega ^{^l}_{_{\ell \ell }}\Bigr ) \Bigl (\varsigma _{_\ell }\Bigr ) \Bigl (\Re _{_\ell }\Bigr ). \end{aligned} \end{aligned}$$

### NQQR grayscale image representation

In this section, an example illustrating the representation of a $3\times 3$ grayscale image shown in Figure 5 using the NQQR model is presented, where the grayscale value of each of the 9 pixels is shown on the corresponding pixel. The image is implemented using the NQQR model by initializing 9 qutrits to $\left| 0\right\rangle$ using the formula $q+1+2n$, where $q=6$ and $n=1$. Among these, $q=6$ qutrits are used to represent the grayscale values, $2\times n=2$ qutrits are used to encode the pixel positions, and 1 ancilla qutrit is employed. Next, $H^{\otimes 2}$ gates are applied on the 2 position qutrits to encode all pixel positions: $\left| 00\right\rangle$, $\left| 01\right\rangle$, $\left| 02\right\rangle$, $\left| 10\right\rangle$, $\left| 11\right\rangle$, $\left| 12\right\rangle$, $\left| 20\right\rangle$, $\left| 21\right\rangle$, $\left| 22\right\rangle$. After that, two-qutrit gates are used to determine each pixel position as Row *Y* and Col *X* and assign its grayscale value.

**Fig. 8:**
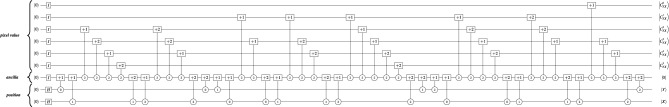
The ternary quantum circuit that represents the implementation of a $3\times 3$ grayscale image shown in Figure 5 using the proposed NQQR model.

The state $\left| \Psi _{_\text {Gray}}\right\rangle$ in Eq (17) represents the $3 \times 3$ grayscale image shown in Figure 5 using the NQQR model. The corresponding quantum circuit for representing this image is shown in Figure 8, with a quantum cost of $QC = 79$. For comparison, implementing the same image using the TNCQR^25^ and QTRQ models^22^ results in ternary quantum circuits with $QC = 149$ and $QC = 203$, respectively. The evolution of the state vector through the NQQR grayscale quantum circuit shown in Figure 8 is provided in Section S1 of the Supplementary Materials.

17 $$\begin{aligned} \begin{aligned} \left| \Psi _{_\text {Gray}}\right\rangle&=\frac{1}{3}\Bigl (&\left| 0\right\rangle \left| 0\right\rangle _{aux}\left| 00\right\rangle+ & \left| 50\right\rangle \left| 0\right\rangle _{aux}\left| 01\right\rangle+ & \left| 75\right\rangle \left| 0\right\rangle _{aux}\left| 02\right\rangle&+\\ & &\left| 90\right\rangle \left| 0\right\rangle _{aux}\left| 10\right\rangle+ & \left| 105\right\rangle \left| 0\right\rangle _{aux}\left| 11\right\rangle+ & \left| 125\right\rangle \left| 0\right\rangle _{aux}\left| 12\right\rangle&+\\ & &\left| 150\right\rangle \left| 0\right\rangle _{aux}\left| 20\right\rangle+ & \left| 225\right\rangle \left| 0\right\rangle _{aux}\left| 21\right\rangle+ & \left| 255\right\rangle \left| 0\right\rangle _{aux}\left| 22\right\rangle \Bigr )\\&=\frac{1}{3}\Bigl (&\left| 000000\right\rangle \left| 0\right\rangle _{aux}\left| 00\right\rangle+ & \left| 001212\right\rangle \left| 0\right\rangle _{aux}\left| 01\right\rangle+ & \left| 002210\right\rangle \left| 0\right\rangle _{aux}\left| 02\right\rangle&+\\ & &\left| 010100\right\rangle \left| 0\right\rangle _{aux}\left| 10\right\rangle+ & \left| 010220\right\rangle \left| 0\right\rangle _{aux}\left| 11\right\rangle+ & \left| 011122\right\rangle \left| 0\right\rangle _{aux}\left| 12\right\rangle&+\\ & &\left| 012120\right\rangle \left| 0\right\rangle _{aux}\left| 20\right\rangle+ & \left| 022100\right\rangle \left| 0\right\rangle _{aux}\left| 21\right\rangle+ & \left| 100110\right\rangle \left| 0\right\rangle _{aux}\left| 22\right\rangle \Bigr ) \end{aligned} \end{aligned}$$

### NQQR complexity analysis

This section presents the calculations used to analyze the performance of the proposed model, including its quantum cost and time complexity. The total quantum cost (${QC}_t$) of the NQQR model is calculated using the formula shown in Eq (18),

18 $$\begin{aligned} {QC}_t=2n+\sum _{Y=0}^{3^n-1} 2 \times (\Re _{_{Y}})_{_{QC}}+3^n\sum _{X=0}^{3^n-1} 2 \times (\varsigma _{_{X}})_{_{QC}}+5, \end{aligned}$$

where $(\Re _{_{Y}})_{_{QC}}$ and $(\varsigma _{_{X}})_{_{QC}}$ represent the QC of each generalized *N*-qutrit gate corresponding to the row or column position for each pixel in a $3^n\times 3^n$ image. The model has recorded a time complexity $O(n3^{2n})$ as discussed in the following Lemma 1, Lemma 2, Lemma 3 and Theorem 1.

#### Lemma 1

The time complexity of preparing pixel positions is *O*(1).

#### Proof

Since the $U_1$ operator prepares $3^{2n}$ pixel positions by applying 2*n* ternary *H* gates on 2*n* qutrits, its time complexity is constant. Hence, the time complexity is *O*(1).

$$\square$$

#### Lemma 2

The time complexity of the quantum operator $U_{_{YX}}$, which sets the grayscale value for one row of pixels is $O(n3^n)$.

#### Proof

The main sub-operation in $U_{_{YX}}$ is $\varpi _{_{YX}}$, which is decomposed into several sub-operations. It begins with $\Re _{_Y}$, a generalized *N*-qutrit gate with *n* control qutrits and 1 target qutrit ($(n+1)$-qutrit M-S gate). For each row, there are $3^n$ columns, each associated with a corresponding $\varsigma _{_X}$ operation, which is also a generalized $(n+1)$-qutrit M-S gate. The operation $\Omega _{_{YX}}$ consists of up to 5 two-qutrit M-S gates to assign the grayscale value for pixel (Y,X). Therefore, for each $U_{_{YX}}$ operator, the total number of required gates includes: 2 $\times$ ($(n+1)$-qutrit M-S gate) for the Row index + $3^n$ $\times$ [ 2 $\times$ ($(n+1)$-qutrit M-S gate) for each Column index + 5 two-qutrit gates for the grayscale value of each pixel ].

Each generalized $(n+1)$-qutrit M-S gate can be decomposed into $4n-3$ two-qutrit M-S gates with $n-1$ ancilla qutrits, in addition to 2*k* one-qutrit gates, where *k* is the number of controls not in state $\left| 2\right\rangle$ and $k \in \{0,1,2\}$. Therefore, the time complexity of each generalized $(n+1)$-qutrit M-S gate is *O*(*n*). Consequently, the time complexity of $U_{YX}$ is $O(n) + O(n3^n)$, which results in $O(n3^n)$. $\square$

#### Lemma 3

The time complexity of $U_2$ quantum operator, which assigns the grayscale values for all $3^{2n}$ pixels, is $O(n3^{2n})$.

#### Proof

The $U_2$ operator iterates over $3^n$ rows and each row iterates over $3^n$ columns using $U_{_{YX}}$ operator to assign the grayscale values for all $3^{2n}$ pixels. According to Lemma 2, the time complexity of a single $U_{_{YX}}$ operation is $O(n3^n)$. Therefore, the time complexity of $U_2$ is $O(3^n)\times O(n3^{n})=O(n3^{2n})$. $\square$

#### Theorem 1

The time complexity of representing $3^n\times 3^n$ grayscale quantum image using the NQQR model in ternary system is $O(n3^{2n})$.

#### Proof

The time complexity of quantum image representation in the NQQR model is calculated based on the quantum operators used in preparing the position information and the grayscale values ($U_1, U_{YX}, U_2$). According to Lemma 1, the time complexity of preparing the pixel position is *O*(1). The step of assigning the grayscale value for each pixel is divided into $3^{n}$ row-wise sub-operations to assign the grayscale value for each pixel. Since the main operation in this step is $U_{_{YX}}$ with time complexity $O(n3^n)$, then time complexity for setting the grayscale value for every pixel ($3^{2n}$ pixels) is $O(n3^{2n})$, as proved in Lemmas 2 and 3. Therefore, the time complexity of representing a $3^n \times 3^n$ grayscale image in the NQQR model is $O(1) + O(n3^{2n})$, which results $O(n3^{2n})$. $\square$

## The proposed NQQR model for RGB images

**Fig. 9:**
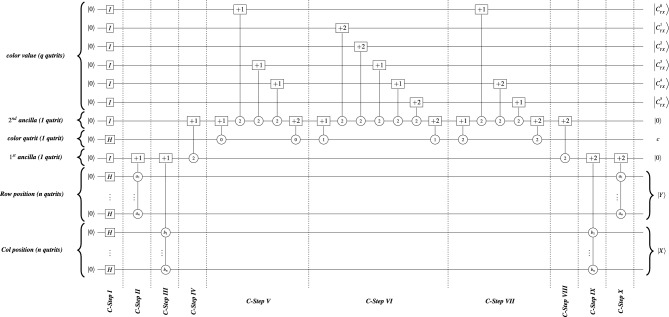
The general ternary quantum circuit for representing a $3^n\times 3^n$ RGB image using the NQQR model.

This section presents the steps (C-Steps) for representing RGB digital images using the proposed NQQR model in ternary quantum system. To represent a $3^n \times 3^n$ RGB image, $2n + q + 3$ qutrits are required. The 2*n* qutrits are used to encode the pixel positions, while $q = 6$ qutrits are used to encode each color value in the range $[0-255]$. An additional color qutrit differentiates between the 3 color channels in superposition, where the states $\left| 0\right\rangle , \left| 1\right\rangle$ and $\left| 2\right\rangle$ correspond to the Red, Green and Blue channels, respectively. The $1^{\text {st}}$ ancilla qutrit is conditionally modified after determining the pixel position (*Y*, *X*) as Row *Y* and Col *X*. After that, the $2^{\text {nd}}$ ancilla qutrit is conditionally modified according to the state of the $1^{st}$ ancilla and the color qutrit to assign the RGB values to each pixel. Figure 9 illustrates the general ternary quantum circuit for representing RGB images using the NQQR model.

- **C-Step I**: Initialize the $2n+q+3$ qutrits to $\left| 0\right\rangle$, then apply $H^{\otimes (2n+1)}$ gates on the 2*n* position qutrits and the color qutrit.
- **C-Step II**: A generalized *N*-qutrit gate with *n* control qutrits (row position *Y*) and a $[+1]$ target gate is used to update the $1^{st}$ ancilla qutrit from $\left| 0\right\rangle$ to $\left| 1\right\rangle$ (Enable row *Y*).
- **C-Step III**: A generalized *N*-qutrit gate with *n* control qutrits (column position *X*) and a $[+1]$ target gate is used to update the $1^{st}$ ancilla qutrit from $\left| 1\right\rangle$ to $\left| 2\right\rangle$ (Enable column *X*).
- **C-Step IV**: A two-qutrit gate, with the $1^{st}$ ancilla as the control qutrit in state $\left| 2\right\rangle$, updates the $2^{nd}$ ancilla from $\left| 0\right\rangle$ to $\left| 1\right\rangle$ using a $[+1]$ target gate. This operation enables the selected pixel (*Y*, *X*), enabling the assignment of its RGB channel values (Enable pixel (*Y*, *X*)).
- **C-Step V**: A two-qutrit gate detects the Red color when the control on the color qutrit is in state $\left| 0\right\rangle$. The target $[+1]$ gate updates the $2^{nd}$ ancilla from $\left| 1\right\rangle$ to $\left| 2\right\rangle$. Then, the Red value is assigned using up to 5 two-qutrit gates from the $2^{nd}$ ancilla to the *q* qutrits. Finally, another two-qutrit gate with control $\left| 0\right\rangle$ and target $[+2]$ disables the Red color and returns the $2^{nd}$ ancilla to $\left| 1\right\rangle$.
- **C-Step VI**: A two-qutrit gate detects the Green color when the control on the color qutrit is in state $\left| 1\right\rangle$. The target $[+1]$ gate updates the $2^{nd}$ ancilla from $\left| 1\right\rangle$ to $\left| 2\right\rangle$. Then, the Green value is assigned using up to 5 two-qutrit gates from the $2^{nd}$ ancilla to the *q* qutrits. Finally, another two-qutrit gate with control $\left| 1\right\rangle$ and target $[+2]$ disables the Green color and returns the $2^{nd}$ ancilla to $\left| 1\right\rangle$.
- **C-Step VII**: A two-qutrit gate detects the Blue color when the control on the color qutrit is in state $\left| 2\right\rangle$. The target $[+1]$ gate updates the $2^{nd}$ ancilla from $\left| 1\right\rangle$ to $\left| 2\right\rangle$. Then, the Blue value is assigned using up to 5 two-qutrit gates from the $2^{nd}$ ancilla to the *q* qutrits. Finally, another two-qutrit gate with control $\left| 2\right\rangle$ and target $[+2]$ disables the Blue color and returns the $2^{nd}$ ancilla to $\left| 1\right\rangle$.
- **C-Step VIII**: A two-qutrit gate, where the control qutrit ($1^{st}$ ancilla) is in state $\left| 2\right\rangle$, reverses the effect of C-Step IV, returning the $2^{nd}$ ancilla from $\left| 1\right\rangle$ to $\left| 0\right\rangle$ using a $[+2]$ target gate (Disable pixel (*Y*, *X*)).
- **C-Step IX**: A generalized *N*-qutrit gate with *n* control qutrits (column position *X*) and a $[+2]$ target gate is used to reverse the effect of C-Step III, returning the $1^{st}$ ancilla from $\left| 2\right\rangle$ to $\left| 1\right\rangle$, preparing the representation for the next pixel in the same row *Y* (Disable column *X*).
- *Repeat C-Steps* $\MakeUppercase {III}-\MakeUppercase {IX}$ *to assign the RGB values for all pixels in the same row.*
- **C-Step X**: A generalized *N*-qutrit gate with *n* control qutrits (row position *Y*) and a $[+2]$ target gate is used to reverse the effect of C-Step II, returning the $1^{st}$ ancilla from $\left| 1\right\rangle$ to $\left| 0\right\rangle$, preparing the representation for the next row of pixels (Disable row *Y*).
- *C-Steps* $\MakeUppercase {II}-\MakeUppercase {X}$ *are repeated to represent all pixels in the image.*

**Fig. 10:**
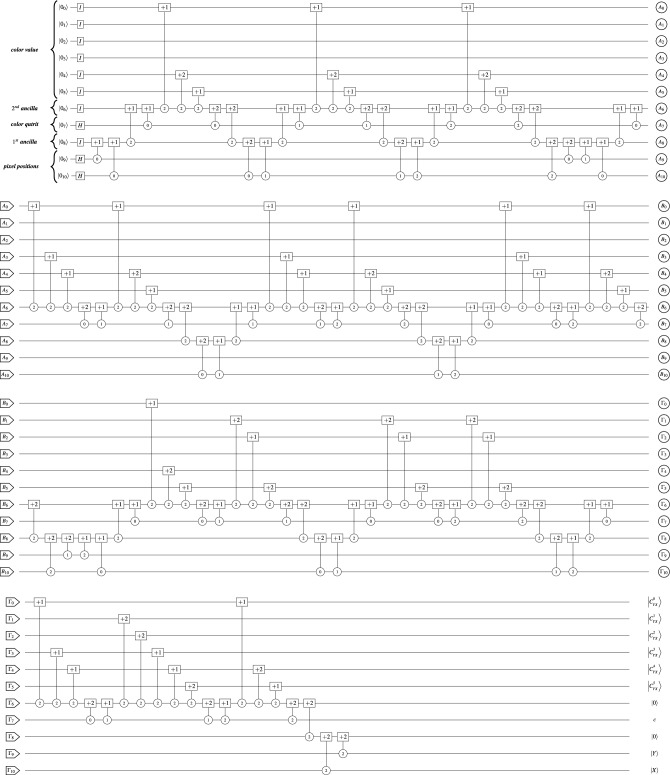
Quantum circuit for the NQQR representation of a $3\times 3$ RGB image shown in Figure 6.

Figure 6 shows a $3\times 3$ RGB image with the color value for each pixel in rage $[0-255]$. To represent this image using the proposed NQQR model, set $q=6$ and $n=1$, resulting in 11 qutrits in the initial state $\left| \Psi _0\right\rangle$, all initialized to $\left| 0\right\rangle$. Then, $H^{\otimes 3}$ gates are applied on the 2 position qutrits and 1 color qutrit. After that, two-qutrit gates are used to determine each pixel position as Row *Y* and Col *X* and assign its RGB color value, as shown in the implemented circuit in Figure 10 with $QC=203$. For comparison, implementing the same image using the TNCQR ^25^ and QTRQ models ^22^ results in ternary quantum circuits with $QC=267$ and $QC=637$, respectively. The corresponding $\left| \Psi _{_\text {RGB}}\right\rangle$ state for this RGB image is shown in Eq (19), while the evolution of the state vector through the NQQR RGB quantum circuit shown in Figure 10 is provided in Section S2 of the Supplementary Materials.

19 $$\begin{aligned} \begin{aligned} \left| \Psi _{_\text {RGB}}\right\rangle =\frac{1}{3\sqrt{3}}&(\left| 100021\right\rangle \left| 0\right\rangle _R+\left| 000000\right\rangle \left| 1\right\rangle _G+\left| 000000\right\rangle \left| 2\right\rangle _B) \left| 0\right\rangle _{{aux}_2} \left| 0\right\rangle _{{aux}_1} \left| 00\right\rangle +\\&(\left| 000000\right\rangle \left| 0\right\rangle _R+\left| 100021\right\rangle \left| 1\right\rangle _G+\left| 000000\right\rangle \left| 2\right\rangle _B) \left| 0\right\rangle _{{aux}_2} \left| 0\right\rangle _{{aux}_1} \left| 01\right\rangle +\\&(\left| 000000\right\rangle \left| 0\right\rangle _R+\left| 000000\right\rangle \left| 1\right\rangle _G+\left| 100021\right\rangle \left| 2\right\rangle _B) \left| 0\right\rangle _{{aux}_2} \left| 0\right\rangle _{{aux}_1} \left| 02\right\rangle +\\&(\left| 100110\right\rangle \left| 0\right\rangle _R+\left| 100021\right\rangle \left| 1\right\rangle _G+\left| 000000\right\rangle \left| 2\right\rangle _B) \left| 0\right\rangle _{{aux}_2} \left| 0\right\rangle _{{aux}_1} \left| 10\right\rangle +\\&(\left| 000000\right\rangle \left| 0\right\rangle _R+\left| 100110\right\rangle \left| 1\right\rangle _G+\left| 100021\right\rangle \left| 2\right\rangle _B) \left| 0\right\rangle _{{aux}_2} \left| 0\right\rangle _{{aux}_1} \left| 11\right\rangle +\\&(\left| 100110\right\rangle \left| 0\right\rangle _R+\left| 000000\right\rangle \left| 1\right\rangle _G+\left| 100021\right\rangle \left| 2\right\rangle _B) \left| 0\right\rangle _{{aux}_2} \left| 0\right\rangle _{{aux}_1} \left| 12\right\rangle +\\&(\left| 100021\right\rangle \left| 0\right\rangle _R+\left| 021002\right\rangle \left| 1\right\rangle _G+\left| 000000\right\rangle \left| 2\right\rangle _B) \left| 0\right\rangle _{{aux}_2} \left| 0\right\rangle _{{aux}_1} \left| 20\right\rangle +\\&(\left| 021002\right\rangle \left| 0\right\rangle _R+\left| 000000\right\rangle \left| 1\right\rangle _G+\left| 021002\right\rangle \left| 2\right\rangle _B) \left| 0\right\rangle _{{aux}_2} \left| 0\right\rangle _{{aux}_1} \left| 21\right\rangle +\\&(\left| 100110\right\rangle \left| 0\right\rangle _R+\left| 022112\right\rangle \left| 1\right\rangle _G+\left| 100021\right\rangle \left| 2\right\rangle _B) \left| 0\right\rangle _{{aux}_2} \left| 0\right\rangle _{{aux}_1} \left| 22\right\rangle . \end{aligned} \end{aligned}$$

## NQQR optimization method

The NQQR model is optimized using the algorithm proposed in^22,24,25^, which consists of two main phases. Phase I (Gate Decomposition) focuses on decomposing each generalized *N*-qutrit gate, where $N \ge 2$, into its simplest form using elementary gates, as described in Section Preliminaries^25^. Phase II (Circuit Simplification) applies gate movement, elimination, and merging rules to reduce the overall circuit cost^22,25^.

### Applying NQQR optimization on grayscale images

**Fig. 11:**
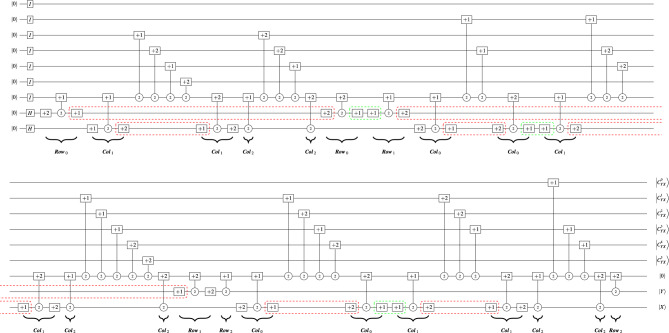
Phase I decomposition of the NQQR quantum circuit shown in Figure 8.

Phase I decomposition of the $3 \times 3$ NQQR grayscale quantum circuit shown in Figure 8 is illustrated in Figure 11, where each Row and Col gates are decomposed to its simplest form.

**Fig. 12:**
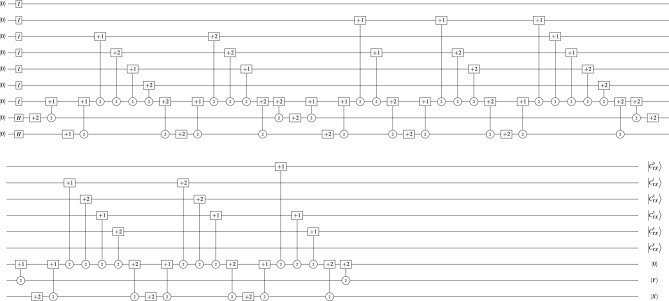
Phase II decomposition of the NQQR quantum circuit shown in Figure 8.

Phase II decomposition is presented in Figure 12. In this phase, redundant (green boxes) or equivalent (red boxes) elementary gates in Figure 11 are merged or eliminated, resulting in an optimized quantum circuit with a total quantum cost $QC = 62$.

**Fig. 13:**
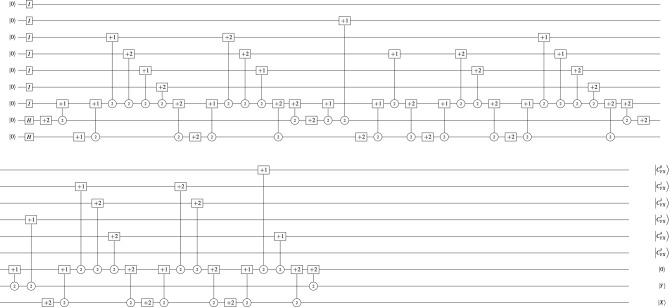
The optimized quantum circuit for the NQQR representation of a $3\times 3$ grayscale image (shown in Figure 5), with a total quantum cost $QC=58$.

The circuit cost is further reduced by combining gates that share the same control *Y* and target *Z*^22,25^. This is achieved for $\left| C^{^1}_{_{YX}}\right\rangle$ with $Y = 1$, $X = 0, 1, 2$, and $Z = [+1]$, and for $\left| C^{^3}_{_{YX}}\right\rangle$ with $Y = 2$, $X = 0, 1, 2$, and $Z = [+1]$, as shown in Eq (20). In each case, the 3 two-qutrit gates are replaced by a single two-qutrit gate with the corresponding control *Y* and target *Z*. This gate is then decomposed into its elementary components, resulting in a fully optimized quantum circuit with a total quantum cost $QC = 58$, as shown in Figure 13.

20 $$\begin{aligned} \begin{aligned} \left| C^{^0}_{_{YX}}\right\rangle&=Z(+1)Y^2X^2\\ \left| C^{^1}_{_{YX}}\right\rangle&=Z(+1)Y^1X^0+Z(+1)Y^1X^1+Z(+1)Y^1X^2+Z(+1)Y^2X^0+Z(+2)Y^2X^1\\&=Z(+1)Y^1+Z(+1)Y^2X^0+Z(+2)Y^2X^1\\ \left| C^{^2}_{_{YX}}\right\rangle&=Z(+1)Y^0X^1+Z(+1)Y^1X^2+Z(+2)Y^0X^2+Z(+2)Y^2X^0+Z(+2)Y^2X^1\\ \left| C^{^3}_{_{YX}}\right\rangle&=Z(+1)Y^1X^0+Z(+1)Y^1X^2+Z(+1)Y^2X^0+Z(+1)Y^2X^1+Z(+1)Y^2X^2+Z(+2)Y^0X^1+Z(+2)Y^0X^2+Z(+2)Y^1X^1\\&=Z(+1)Y^1X^0+Z(+1)Y^1X^2+Z(+1)Y^2+Z(+2)Y^0X^1+Z(+2)Y^0X^2+Z(+2)Y^1X^1\\ \left| C^{^4}_{_{YX}}\right\rangle&=Z(+1)Y^0X^1+Z(+1)Y^0X^2+Z(+1)Y^2X^2+Z(+2)Y^1X^1+Z(+2)Y^1X^2+Z(+2)Y^2X^0+Z(+2)Y^0X^2+Z(+2)Y^1X^1\\ \left| C^{^5}_{_{YX}}\right\rangle&=Z(+1)Y^0X^1+Z(+1)Y^1X^2 \end{aligned} \end{aligned}$$

### Applying NQQR optimization on RGB images

**Fig. 14:**
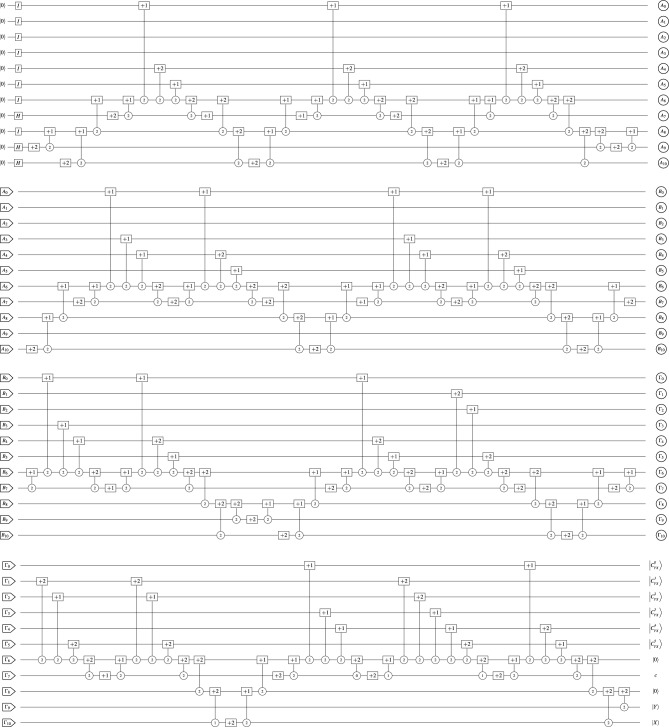
The optimized quantum circuit for the NQQR representation of a $3\times 3$ RGB image (shown in Figure 6), with a total quantum cost $QC=158$.

Phase I decomposed each two-qutrit gate in Figure 10 to its elementary gates. Phase II then applies the gate elimination and merging rules, reducing the overall quantum cost to $QC = 158$. The optimized RGB quantum circuit obtained after applying Phases I and II is shown in Figure 14.

## Experimental results

This section presents a comparative evaluation of the proposed NQQR model against existing quantum image representation models, including ternary models such as TNCQR^25^ and QTRQ^22^, as well as binary models such as FRQI^16^, NEQR^17^, MCQI^18^ and NCQI ^19^. The evaluation is conducted analytically based on quantum cost (QC) and circuit complexity for representing $3^n \times 3^n$ grayscale and RGB images in ternary quantum systems, which are standard metrics in quantum circuit design. The proposed model has been implemented and evaluated using Google Cirq^37^ to verify the correctness of the circuit construction and state evolution, as well as to analyze circuit behavior and estimate computational characteristics.

**Table 2:** Comparison between the proposed NQQR model with FRQI^16^, MCQI^18^, NEQR^17^, NCQI^19^, QTRQ^22,25^ and TNCQR^25^ models in terms of total quantum cost and time complexity.

Model	Image Size	Image mode	Qudit type	Quantum Cost (*QC*)	Time Complexity
FRQI^16^	$2^n\times 2^n$	Grayscale	qubit	$QC_{_1}^{^\text {Gray}}=2n+2^{4n}-3\times 2^{2n}$	$O(2^{4n})$
MCQI^18^	$2^n\times 2^n$	RGB	qubit	$QC_{_1}^{^\text {RGB}}=2n+3\times 2^{4n}-9\times 2^{2n}$	$O(2^{4n})$
NEQR^17^	$2^n\times 2^n$	Grayscale	qubit	$QC_{_2}^{^\text {Gray}}=2n+\sum _{Y=0}^{2^n-1}\sum _{X=0}^{2^n-1}8\times (YX)_{QC}$	$O(qn2^{2n})$
NCQI^19^	$2^n\times 2^n$	RGB	qubit	$QC_{_2}^{^\text {RGB}}=2n+\sum _{Y=0}^{2^n-1}\sum _{X=0}^{2^n-1}24\times (YX)_{QC}$	$O(6qn2^{2n})$
QTRQ^22,25^	$3^n\times 3^n$	Grayscale	qutrit	$QC_{_3}^{^\text {Gray}}=2n+\sum _{Y=0}^{3^n-1}\sum _{X=0}^{3^n-1}5\times (YX)_{QC}$	$O(qn3^{2n})$
		RGB		$QC_{_3}^{^{\text {RGB}}}=2n+1+ \sum _{Y=0}^{3^n-1}\sum _{X=0}^{3^n-1} (5 \times (YX)_{R} + 5 \times (YX)_{G} + 5 \times (YX)_{B})$	
TNCQR^25^	$3^n\times 3^n$	Grayscale	qutrit	$QC_{_4}^{^\text {Gray}}=2n+\sum _{Y=0}^{3^n-1}\sum _{X=0}^{3^n-1}(2\times (YX)_{QC}+5)$	$O(n3^{2n})$
		RGB		$QC_{_4}^{^{\text {RGB}}}=2n+1+\sum _{Y=0}^{3^n-1}\sum _{X=0}^{3^n-1} (2\times (YX)_{QC} + 29)$	
NQQR	$3^n\times 3^n$	Grayscale	qutrit	$QC_{_5}^{^\text {Gray}}=2n+\sum _{Y=0}^{3^n-1} 2\times \Re _{QC}+3^n\sum _{X=0}^{3^n-1}(2\times \varsigma _{_{QC}}+5)$	$O(n3^{2n})$
		RGB		$QC_{_5}^{^{\text {RGB}}}=2n+1+\sum _{Y=0}^{3^n-1} 2\times \Re _{QC}+3^n\sum _{X=0}^{3^n-1}(2\times \varsigma _{_{QC}}+31)$	

Table 2 summarizes the comparison terms among the compared models, including the equations used to calculate the total quantum cost of representing $3^n \times 3^n$ grayscale and RGB images for each model in Tables 3 and 4, respectively, as well as the time complexity. In terms of time complexity, the binary FRQI model records $O(2^{4n})$ for representing grayscale images, while the NEQR records $O(qn2^{2n})$. For RGB images, the binary MCQI model records $O(2^{4n})$, while the NCQI records $O(6qn2^{2n})$. The ternary QTRQ model records $O(qn3^{2n})$ for representing grayscale or RGB images with a color range of $3^q$, where $q=6$. The ternary TNCQR model achieves $O(n3^{2n})$ for representing a $3^n \times 3^n$ grayscale or RGB image, which is the same time complexity achieved by the proposed NQQR model. The proposed NQQR model has a time complexity of $O(n3^{2n})$, which is higher than qubit-based models due to the richer structure of qutrits, where each unit encodes three levels instead of two. This increased complexity reflects higher information density rather than reduced efficiency. Additionally, the model improves scalability for grayscale and RGB image representation by reducing redundant encoding operations.

To illustratively evaluate quantum cost efficiency, the $3 \times 3$ grayscale image shown in Figure 5 was encoded using different ternary quantum image representation models. The proposed NQQR model represents the image using 9 qutrits with an initial quantum cost of $QC = 79$, which is reduced to $QC = 58$ after optimization. The TNCQR model also uses 9 qutrits, with an initial quantum cost of $QC = 149$, reduced to $QC = 85$ after optimization^25^. The QTRQ model uses 8 qutrits with an initial quantum cost of $QC = 203$, which is reduced to $QC = 107$ after optimization^22^. While this example focuses on ternary representations to clarify the encoding mechanism, a comprehensive comparison with both binary/ternary-based models across different image sizes is provided in Table 3.

**Table 3:** Comparison of the proposed NQQR model with FRQI^16^, NEQR^17^, QTRQ^22^ and TNCQR^25^ models for representing $3^{n_{\text {qutrit}}} \times 3^{n_{\text {qutrit}}}$ grayscale images, in terms of the total number of qudits (*M*) required by each model and the total quantum cost (*QC*) under the worst-case scenario.

$n_{\text {qutrit}}$	Image Size	$n_{\text {qubit}}$	FRQI^16^		NEQR^17^		QTRQ^22,25^		TNCQR^25^			NQQR		
	$(3^{n_{\text {qutrit}}} \times 3^{n_{\text {qutrit}}})$		$M_{_1}^{^\text {Gray}}$	$QC_{_1}^{^\text {Gray}}$	$M_{_2}^{^\text {Gray}}$	$QC_{_2}^{^\text {Gray}}$	$M_{_3}^{^\text {Gray}}$	$QC_{_3}^{^\text {Gray}}$	$M_{_4}^{^\text {Gray}}$	$QC_{_4}^{^\text {Gray}}$	$QC_{_4}^{^\text {Gray}}$_optimized_	$M_{_5}^{^\text {Gray}}$	$QC_{_5}^{^\text {Gray}}$	$QC_{_5}^{^\text {Gray}}$_optimized_
1	$3\times 3$	2	5	212	12	2,092	8	347	9	185	113	9	103	83
2	$9\times 9$	4	9	64,776	16	329,840	10	7,429	11	3,379	2,635	11	1,789	1,069
3	$27\times 27$	5	11	1,045,514	18	11,926,450	12	105,711	13	45,933	35,361	13	23,307	18,351
4	$81\times 81$	7	15	268,386,318	22	1,719,769,334	14	1,301,273	15	553,319	423,191	15	276,353	215,345
5	$243\times 243$	8	17	4,294,770,704	24	61,915,947,064	16	14,860,675	17	6,239,521	4,753,453	17	3,101,743	2,399,755
6	$729\times 729$	10	21	1,099,508,482,068	28	8,916,087,693,692	18	162,089,517	19	67,493,019	51,284,067	19	33,523,077	25,805,517
7	$2187\times 2187$	12	25	281,474,926,379,032	32	1,283,918,349,757,632	20	1,713,897,239	21	709,473,749	538,084,025	21	352,495,571	270,347,111

Table 3 presents the total quantum cost for representing $3^{n_{\text {qutrit}}} \times 3^{n_{\text {qutrit}}}$ grayscale images with $n_{\text {qutrit}} \in \{1,2,\dots ,7\}$ under a worst-case scenario, assuming that 5 ternary digits are modified to encode each pixel’s grayscale value. The quantum cost of the NQQR model is calculated using the $QC_{_5}^{^\text {Gray}}$ equation in Table 2, where $n=n_{\text {qutrit}}$ determines the image size, $\Re _{QC}$ and $\varsigma _{QC}$ represents the quantum cost of the *N*-qutrit gates used to encode the row and column positions, respectively. The total number of qutrits required by NQQR model is $M_{_5}^{^\text {Gray}}=2 \times n_{\text {qutrit}}+q+1$, where $q=6$ qutrits for ternary models. The quantum costs of the TNCQR and QTRQ models are computed using the equations $QC_{_4}^{^\text {Gray}}$ and $QC_{_3}^{^\text {Gray}}$, respectively, where $(YX)_{QC}$ represents the quantum cost of the *N*-qutrit gate used to encode the position of the pixel (*Y*, *X*). The total number of qutrits required by each model is $M_{_2}^{^\text {Gray}}=2 \times n_{\text {qutrit}}+q+1$ for the TNCQR model and $M_{_1}^{^\text {Gray}}=2 \times n_{\text {qutrit}}+q$ for the QTRQ model. For binary-based models, the quantum cost of the NEQR and the FRQI models is calculated using the equations $QC_{_2}^{^\text {Gray}}$ and $QC_{_1}^{^\text {Gray}}$, respectively, where $n=n_{\text {qubit}}=\lceil \log _{2}(3^{n_{\text {qutrit}}}) \rceil$ which represents the required number of qubits to represent $3^{n_{\text {qutrit}}} \times 3^{n_{\text {qutrit}}}$ image. The total number of qubits required by each model is $M_{_2}^{^\text {Gray}}=2 \times n_{\text {qubit}}+q$ and $M_{_1}^{^\text {Gray}}=2 \times n_{\text {qubit}}+1$, where $q=8$ qubits. These results demonstrate the efficiency of the proposed NQQR model in terms of both number of qudits (qubits or qutrits) required and the quantum cost. By comparing the proposed model with the ternary-based models (QTRQ and TNCQR), the proposed model is nearly close to the number of qutrits used (*M*) and shows a significant improvement in the quantum cost (*QC*), particularly as the image size increases. By comparing the proposed model with the binary-based models (FRQI and NEQR), the proposed model requires less number of qudits and significant improvement in the quantum cost as the image size increases.

In order to present the achieved improvement more clearly, the NQQR model ($QC_{_5}^{^\text {Gray}}$) achieves quantum cost improvement of $30.52\%$, $48.8\%$, $77.26\%$, $99.19\%$ and $92.89\%$ compared to the optimized TNCQR ($QC_{_4}^{^\text {Gray}}$_optimized_), the original TNCQR ($QC_{_4}^{^\text {Gray}}$), the QTRQ ($QC_{_3}^{^\text {Gray}}$), the NEQR ($QC_{_2}^{^\text {Gray}}$), the FRQI models ($QC_{_1}^{^\text {Gray}}$), respectively. Furthermore, the optimized NQQR model ($QC_{_5}^{^\text {Gray}}$_optimized_) achieves additional improvements of $24.57\%$, $47.45\%$, $61.40\%$, $82.85\%$, $99.36\%$ and $93.9\%$ over the original NQQR ($QC_{_5}^{^\text {Gray}}$), the optimized TNCQR ($QC_{_4}^{^\text {Gray}}$_optimized_), the TNCQR ($QC_{_4}^{^\text {Gray}}$) and the QTRQ ($QC_{_3}^{^\text {Gray}}$), the NEQR ($QC_{_2}^{^\text {Gray}}$), the FRQI models ($QC_{_1}^{^\text {Gray}}$), respectively. This comparison demonstrates that the proposed NQQR model is better in quantum cost than the existing models before and after optimization, confirming that its circuit structure is inherently more efficient and more amenable to optimization.

**Table 4:** Comparison of the proposed NQQR model with MCQI ^18^, NCQI ^19^, QTRQ ^22^ and TNCQR ^25^ models for representing $3^{n_{\text {qutrit}}} \times 3^{n_{\text {qutrit}}}$ RGB images, in terms of the total number of qudits (*M*) required by each model and the total quantum cost (*QC*) under the worst-case scenario.

$n_{\text {qutrit}}$	Image Size	$n_{\text {qubit}}$	MCQI ^18^		NCQI ^19^		QTRQ ^22,25^		TNCQR ^25^			NQQR		
	$(3^{n_{\text {qutrit}}} \times 3^{n_{\text {qutrit}}})$		$M_{_1}^{^{\text {RGB}}}$	$QC_{_1}^{^{\text {RGB}}}$	$M_{_2}^{^{\text {RGB}}}$	$QC_{_2}^{^{\text {RGB}}}$	$M_{_3}^{^{\text {RGB}}}$	$QC_{_3}^{^{\text {RGB}}}$	$M_{_4}^{^{\text {RGB}}}$	$QC_{_4}^{^{\text {RGB}}}$	$QC_{_4}^{^{\text {RGB}}}$_optimized_	$M_{_5}^{^{\text {RGB}}}$	$QC_{_5}^{^{\text {RGB}}}$	$QC_{_5}^{^{\text {RGB}}}$_optimized_
1	$3\times 3$	2	7	628	28	6,268	9	1,758	10	402	284	11	338	272
2	$9\times 9$	4	11	194312	32	989,504	11	28,760	12	5,324	4,174	13	3,896	2,770
3	$27\times 27$	5	13	3136522	34	35,779,330	13	375,442	14	63,430	49,212	15	42,262	33,660
4	$81\times 81$	7	17	805158926	38	5,159,307,974	15	4,428,684	16	710,784	547,850	17	446,940	353,126
5	$243\times 243$	8	19	12884312080	40	185,747,841,160	17	49,305,926	18	7,656,698	5,875,384	19	4,637,018	3,639,784
6	$729\times 729$	10	23	3298525446164	44	26,748,263,081,036	19	528,783,808	20	80,247,604	61,381,446	21	47,340,544	36,965,778
7	$2187\times 2187$	12	27	844424779137048	48	3,851,755,049,272,848	21	5,524,329,210	22	824,265,006	628,960,436	23	476,852,766	370,789,460

The RGB image shown in Figure 6 is represented using ternary representations to clarify the encoding mechanism, a comprehensive comparison with both binary/ternary models across different image sizes is provided in Table 4. The NQQR model represents the RGB image using 11 qutrits with an initial quantum cost of $QC = 203$, which is reduced to $QC = 158$ after optimization. The TNCQR model requires 10 qutrits with an initial quantum cost of $QC = 267$, reduced to $QC = 170$ ^25^. The QTRQ model uses 9 qutrits with an initial quantum cost of $QC = 637$, without optimization ^25^.

Table 4 presents the total quantum cost under a worst-case scenario for representing $3^{n_{\text {qutrit}}} \times 3^{n_{\text {qutrit}}}$ RGB images using the ternary NQQR, TNCQR, QTRQ models, as well as the binary MCQI and the NCQI models, along with the total number of qudits (qutrits or qubits) required by each model. The quantum cost for the NQQR model is calculated using the $QC_{_5}^{^{\text {RGB}}}$ equation in Table 2 with $M_{_5}^{^{\text {RGB}}}=2\times n_{\text {qutrit}} +q+3$ qutrits, where $q=6$. The quantum cost for the TNCQR model is computed using the $QC_{_4}^{^{\text {RGB}}}$ equation with $M_{_4}^{^{\text {RGB}}}=2\times n_{\text {qutrit}}+q+2$ qutrits, while the quantum cost for the QTRQ model is calculated using the $QC_{_3}^{^{\text {RGB}}}$ equation with $M_{_3}^{^{\text {RGB}}}=2\times n_{\text {qutrit}}+q+1$ qutrits, where $(YX)_R$, $(YX)_G$, and $(YX)_B$ are the quantum cost of the *N*-qutrit gates applied to the Red, Green, and Blue channels, respectively. The quantum cost for the NCQI model for representing $3^{n_{\text {qutrit}}} \times 3^{n_{\text {qutrit}}}$ RGB image is calculated using the $QC_{_2}^{^\text {RGB}}$ equation with $M_{_2}^{^{\text {RGB}}}=2\times n_{\text {qubit}}+3q$ qubits, where $q=8$. Moreover, the quantum cost for the MCQI model is calculated using the $QC_{_1}^{^\text {RGB}}$ equation with $M_{_1}^{^{\text {RGB}}}=2\times n_{\text {qubit}}+3$ qubits. As shown in the table, the proposed NQQR model demonstrates a clear reduction in quantum cost (*QC*) compared with the ternary models (TNCQR and QTRQ) and the binary models (MCQI and NCQI), particularly as the image size increases. In addition, by comparing the number of required qudits (qubits or qutrits), the proposed model uses a number of qutrits close to the ternary models and less than the qubits used in the binary models.

A similar evaluation procedure is followed for RGB images. Based on the comparisons presented in Table 4, the proposed NQQR model achieves quantum cost improvement of $12.62\%$, $33.7\%$, $88.41\%$, $99.15\%$ and $91.82\%$ compared to the optimized TNCQR, the original TNCQR, the QTRQ , the NCQI and the MCQI models, respectively. Moreover, the optimized NQQR model further reduces the quantum cost by $22.21\%$, $31.98\%$, $48.43\%$, $90.98\%$, $99.33\%$ and $93.45\%$ compared to the original NQQR, the optimized TNCQR, the original TNCQR, and the QTRQ, the NCQI and the MCQI models, respectively. This confirms that the efficiency and optimization advantages of the NQQR model naturally extend to multi-channel RGB image representation. The improvement arises because each *N*-qutrit gate in the NQQR model is constructed using *n* control qutrits and one target qutrit for both grayscale and RGB images. In contrast, the TNCQR model uses *N*-qutrit gates with 2*n* control qutrits and one target qutrit for both grayscale and RGB images, while the QTRQ model uses 2*n* control qutrits for grayscale images and $2n+1$ control qutrits for RGB images, resulting in higher quantum cost and time complexity. Moreover, the FRQI, the NEQR, the MCQI and the NCQI models use 2n-CNOT gates each with 2*n* control qubits and one target qubit for grayscale and RGB images, respectively, where the quantum cost for each 2n-CNOT gate is calculated according to reversible benchmarks^38^.

An additional point of comparison related to NEQRX scheme^28^, which proposes an encryption algorithm that integrates the NEQR encoding model^17^, a generalized affine transformation for scrambling pixel positions, and a logistic map to modify pixel intensity values^39^. In addition, NEQRX presents circuit-level optimization techniques for NEQR-based circuits representing a $2 \times 2$ grayscale image, leading to reductions in both quantum cost and time complexity compared to the original NEQR model.

In this study, the proposed NQQR model is used to represent the same $2 \times 2$ grayscale image at the representation level. The results indicate that ternary image representation using NQQR achieves a lower quantum cost and reduced time complexity when compared with the NEQR encoding stage prior to encryption, as shown in Table 5. After applying circuit optimization to the NQQR constructed circuit, the quantum cost is further reduced. A slight increase in time complexity is observed due to the decomposition of multi-controlled *N*-qutrit gates into elementary gates, resulting in deeper circuit layers. These results follow a standard circuit-level optimization strategy and highlight the advantage of qutrit-based image representation in enabling more efficient and compact encoding compared to qubit-based approaches.

**Table 5:** Circuit-level comparison between the proposed NQQR representation and NEQR-based encoding optimized in NEQRX ^28^ for a $2\times 2$ grayscale image, in terms of quantum cost and time complexity.

	Circuit	Quantum Cost	Time Complexity
Normal	NEQR	234	147
	NQQR	42	21
After Optimization	NEQR	91	52
	NQQR	28	25

In addition to computational efficiency, it is important to clarify the implications of the proposed representation on image retrieval accuracy. It is important to note that the proposed NQQR model represents image data in a lossless and deterministic manner, where each pixel value is explicitly encoded in the quantum state. Therefore, unlike approximate image processing techniques, no information loss or reconstruction error is introduced. Thus the evaluation focuses on computational efficiency metrics such as quantum cost and time complexity.

Experimental results show that the proposed NQQR model achieves lower quantum cost than comparable ternary and binary quantum image representation models, while exhibiting time complexity comparable to ternary models. Although the NQQR model requires a larger number of elementary gates, this design choice simplifies circuit structures and significantly reduces quantum cost, making it more suitable for scalable grayscale and RGB image representation in ternary quantum systems.

## Performance evaluation of NQQR under quantum noise

This section presents the performance evaluation of the proposed NQQR model under different levels of quantum depolarizing noise using Google Cirq^27,37,40^. The objective of this study is to investigate the reconstruction quality of encoded images in the presence of quantum noise and to evaluate the robustness of the proposed representation against quantum state degradation.

Quantum depolarizing noise is applied to qutrit states to simulate realistic quantum channel imperfections^27,40^. Monte Carlo sampling is employed to approximate the expectation values of noisy quantum measurement outcomes, resulting in more stable estimates of the reconstructed pixel intensities^41,42^. A median-based Monte Carlo estimation is employed to improve the reconstructed image, effectively reducing the impact of quantum noise without relying on quantum error correction techniques. The experiments are conducted on both grayscale and RGB images with multiple Monte Carlo repetitions.

The reconstruction performance is evaluated using Mean Squared Error (MSE) and Peak Signal-to-Noise Ratio (PSNR), computed between the original, noisy, and enhanced images^43–45^. The PSNR metric is computed according to Eq (21)^43–45^. Since grayscale and RGB images differ in the number of channels, the MSE formulation is adapted accordingly for each image representation.

21 $$\begin{aligned} PSNR = 20 \log _{10}\left( \frac{255}{\sqrt{MSE}}\right) \end{aligned}$$

**Fig. 15:**
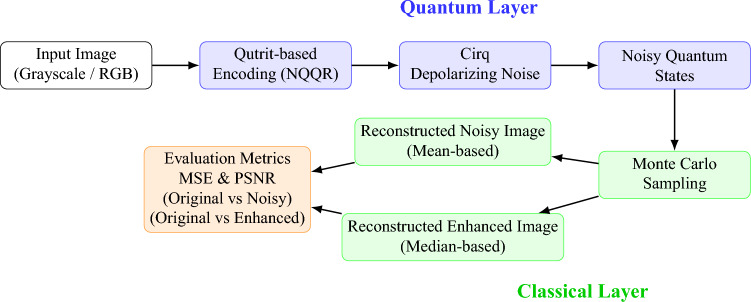
NQQR reconstruction pipeline under quantum depolarizing noise. The blue blocks represent the quantum layer, including image encoding and noise application, while the green blocks represent the classical layer, including Monte Carlo sampling and statistical estimation of pixel intensities. The process yields two reconstructed outputs: a noisy (mean-based) and an enhanced (median-based) image, both evaluated using MSE and PSNR metrics.

The evaluation demonstrates the impact of quantum noise on the encoded image representation and confirms the effectiveness of the proposed statistical reconstruction approach. Complete removal of quantum depolarizing noise is not the objective of this method; instead, the proposed statistical reconstruction combined with median-based processing effectively reduces its impact. Figure 15 illustrates the overall NQQR reconstruction pipeline under quantum depolarizing noise, highlighting the interaction between the quantum processing layer and the classical post-processing stage.

### Grayscale image reconstruction results

The reconstruction performance for grayscale images is evaluated using the standard image quality metrics, MSE and PSNR. The MSE is computed as defined in Eq (22) ^43–45^, which provides a quantitative measure of the reconstruction error between the original and reconstructed images.

22 $$\begin{aligned} MSE = \frac{1}{MN} \sum _{y=0}^{M-1} \sum _{x=0}^{N-1} \left( Img(y,x) - \hat{Img}(y,x) \right) ^2, \end{aligned}$$

where $M=N=3^n$ for the proposed NQQR image representation. *Img*(*y*, *x*) and $\hat{Img}(y,x)$ represent the pixel values at row *y* and column *x* in the original image and the reconstructed image, respectively. Table 6 presents the MSE and PSNR values for the noisy and enhanced reconstructions of the $3\times 3$ grayscale image shown in Figure 5, as well as for the $9\times 9$ and $27\times 27$ grayscale standard Lena image ^46^. This comparison illustrates the performance of the proposed method under different image sizes.

**Table 6:** Comparison of MSE and PSNR values for noisy and enhanced reconstructions of $3\times 3$, $9\times 9$ and $27\times 27$ grayscale images under different levels of depolarizing noise and Monte Carlo repetitions.

Image Size	Image Mode	Repetitions	Noise (%)	$\text {MSE}_{\text {noisy}}$	$\text {PSNR}_{\text {noisy}}$	$\text {MSE}_{\text {enhanced}}$	$\text {PSNR}_{\text {enhanced}}$
$3\times 3$	Grayscale	1000	0	0.0000	$\infty$	0.0000	$\infty$
			10	3399.2222	12.82	0.0000	$\infty$
			20	9212.3704	8.49	3049.6667	13.29
$9\times 9$	Grayscale	3000	0	0.0000	$\infty$	0.0000	$\infty$
			10	1658.5926	15.93	1.6049	46.08
			20	4219.0370	11.88	243.6914	24.26
$27\times 27$	Grayscale	7000	0	0.0000	$\infty$	0.0000	$\infty$
			10	2548.4333	14.06	354.3748	22.63
			20	6313.4125	10.12	1782.4343	15.62

**Fig. 16:**
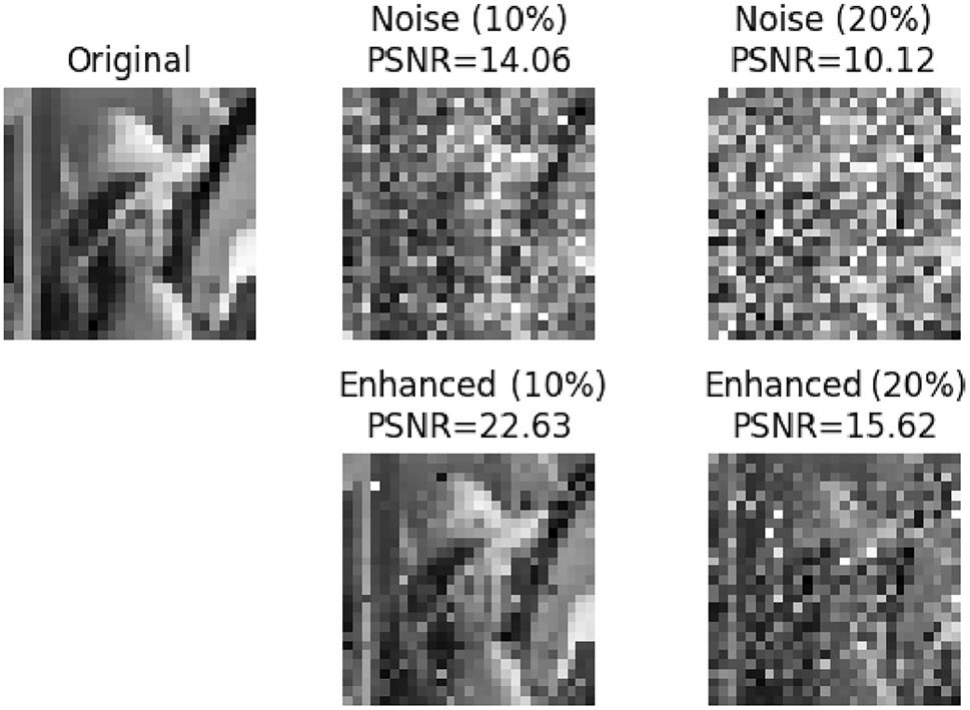
Visual reconstruction results for a $27\times 27$ grayscale Lena image under $10\%$ and $20\%$ depolarizing noise levels.

Figure 16 presents the visual comparison for the $27\times 27$ grayscale version of the standard Lena image between the noisy and enhanced reconstructions at $10\%$ and $20\%$ noise levels.

The results indicate that the enhanced reconstruction significantly improves image quality at low noise levels and continues to provide noticeable improvements over the noisy reconstruction under higher depolarizing noise conditions. This behavior suggests that the proposed statistical reconstruction and median-based processing can effectively approximate the original image across different image sizes under low depolarizing noise levels.

### RGB image reconstruction results

The reconstruction performance for RGB images is evaluated using the same statistical framework adopted for grayscale images, extending the analysis to multi-channel image representations. Similar to the grayscale case, the reconstruction quality is assessed using MSE and PSNR. For RGB images, the MSE is computed by averaging the squared error across all three color channels (R, G, and B), following the formula in Eq (23).

23 $$\begin{aligned} MSE = \frac{1}{3 MN} \sum _{c \in \{R,G,B\}} \sum _{y=0}^{M-1} \sum _{x=0}^{N-1} \left( Img_c(y,x) - \hat{Img}_c(y,x) \right) ^2, \end{aligned}$$

where $M=N=3^n$, $Img_c(y,x)$ and $\hat{Img}_c(y,x)$ represent the pixel values of channel *c* at row *y* and column *x* in the original image and the reconstructed image, respectively. Table 7 presents the MSE and PSNR values for the noisy and enhanced reconstructions of the $3\times 3$ RGB example shown in Figure 6, as well as for the $9\times 9$ and $27\times 27$ RGB standard Lena image ^46^.

**Table 7:** Comparison of MSE and PSNR values for noisy and enhanced reconstructions of $3\times 3$, $9\times 9$ and $27\times 27$ RGB images under different levels of depolarizing noise and Monte Carlo repetitions.

Image Size	Image Mode	Repetitions	Noise (%)	$\text {MSE}_{\text {noisy}}$	$\text {PSNR}_{\text {noisy}}$	$\text {MSE}_{\text {enhanced}}$	$\text {PSNR}_{\text {enhanced}}$
$3\times 3$	RGB	1000	0	0.0000	$\infty$	0.0000	$\infty$
			10	2870.7407	13.55	2.4815	44.18
			20	9286.4074	8.45	4180.6296	11.92
$9\times 9$	RGB	3000	0	0.0000	$\infty$	0.0000	$\infty$
			10	1821.4691	15.53	102.0453	28.04
			20	5426.1111	10.79	1015.6132	18.06
$27\times 27$	RGB	7000	0	0.0000	$\infty$	0.0000	$\infty$
			10	3694.6023	12.45	2409.9333	14.31
			20	6635.2041	9.92	4603.5389	11.50

**Fig. 17:**
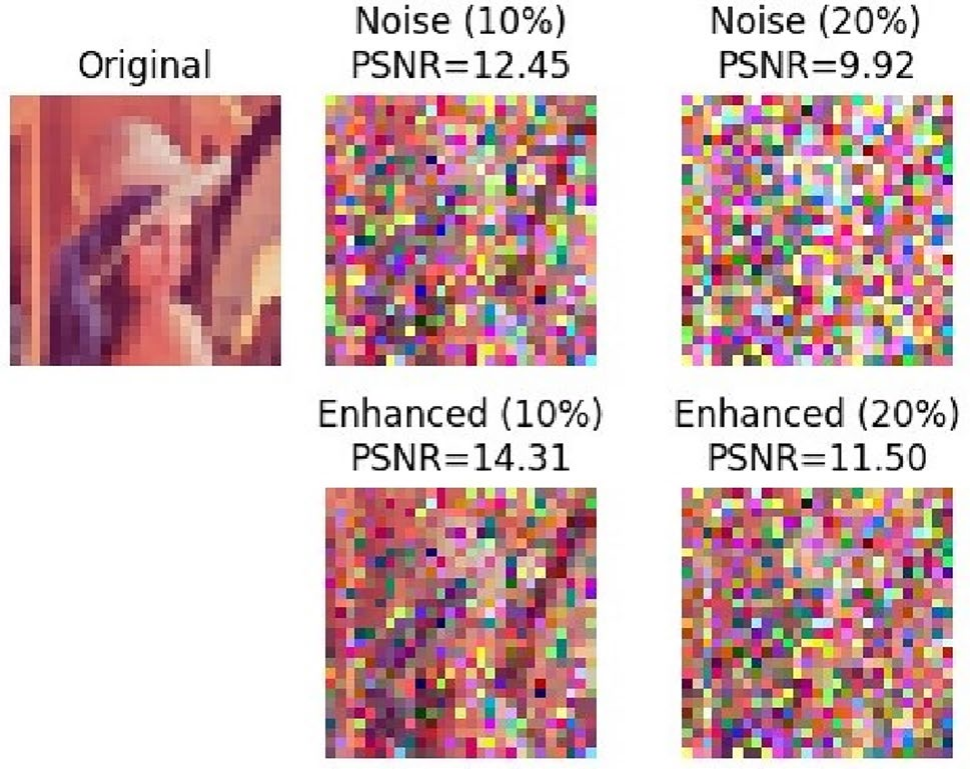
Visual reconstruction results for a $27\times 27$ RGB Lena image under $10\%$ and $20\%$ depolarizing noise levels.

Figure 17 presents the visual comparison for the $27\times 27$ RGB version of the standard Lena image between the noisy and enhanced reconstructions at $10\%$ and $20\%$ noise levels.

The results in this section confirm that the proposed NQQR representation model provides stable and scalable reconstruction performance under quantum noise, with median-based Monte Carlo estimation improves reconstruction quality without relying on quantum error correction techniques.

## Conclusion

Ternary quantum image processing (TQIP) arises from integrating qutrit-based quantum computing with image representation and offers an alternative direction to conventional qubit-based encoding models. In this context, this paper introduced Novel Qutrit Quantum Representation (NQQR) model, a scalable representation model for encoding grayscale and RGB images of size $3^n \times 3^n$ within a ternary quantum system.

The proposed model encodes pixel positions using a superposition of 2*n* qutrits and represents grayscale or color intensity values using 6 qutrits corresponding to the range $[0-255]$. For RGB images, an additional color qutrit is employed to distinguish color channels, while an ancilla qutrit is utilized to simplify multi-controlled *N*-qutrit gates, thereby reducing circuit complexity at the representation level.

Experimental results show that the NQQR model achieves a time complexity of $O(n3^{2n})$ by iteratively encoding all $3^{2n}$ pixel positions and corresponding color intensity row by row. The optimized NQQR formulation further reduces quantum cost through the increased use of elementary one-qutrit and two-qutrit gates. Compared to non-optimized qubit/qutrit-based encoding models, quantum cost reductions of up to $99.36\%$ for grayscale images and $99.33\%$ for RGB images are achieved, while preserving scalability for large ternary quantum systems.

The results presented in this work are implemented and evaluated using Google Cirq for ternary quantum systems, including circuit construction and complexity analysis. This is consistent with most existing quantum image representation studies, due to the current limitations of available quantum hardware for native ternary (qutrit-based) systems. Furthermore, the noise resilience of the encoded images using the proposed model is evaluated under different levels of quantum depolarizing noise. The reconstructed images are obtained using Monte Carlo sampling, and the results demonstrate that the proposed statistical reconstruction approach effectively reduces the impact of quantum noise. In particular, the median-based Monte Carlo estimation significantly improves reconstruction quality across different image sizes and noise levels while maintaining scalability. As quantum technologies continue to evolve, future work can include validation of the proposed model using experimental platforms. While the present work focuses on image representation, future work can also explore the way that ternary encoding can be used for developing quantum image processing algorithms for TQIP, such as encryption or filtering.

## Supplementary Information

Supplementary Material

## Data Availability

The complete Cirq-based implementation of the proposed NQQR representation model, including noise modeling and reconstruction procedures, is publicly available at Zenodo: https://doi.org/10.5281/zenodo.20357973
