# Supplementary material for: NQQR: scalable qutrit image representation
Source: Sci Rep. 2026 Jun 23;16:19535. doi: 10.1038/s41598-026-57304-9 (PMC13291318; doi:10.1038/s41598-026-57304-9)
Supplement: Supplementary file 1 — Supplementary Material [file 41598_2026_57304_MOESM1_ESM.pdf]

Supplementary Materials for NQQR: Scalable Qutrit Image Representation

Mirna Rofail[1], Rasha Montaser[2], Ahmed Younes[1,3]

[1]Department of Mathematics and Computer Science, Faculty of Science, Alexandria University, 21526, Egypt
[2]Department of Information Systems, Faculty of Computers and Information Science, Damanhour University, 22511, Egypt
[3]Faculty of Computer Science and Engineering, Alamein International University, 51718, Egypt

S1 State Vector Evolution of NQQR Grayscale Image

This section presents the state vector evolution of the quantum circuit shown in Figure S1, which implements the 3×3 grayscale image in Figure S2 using the NQQR model. The approach follows the standard formalism described by Nielsen[1] and Yanofsky[2], where quantum states are traced through each gate application. Each intermediate state $|\Phi_i\rangle$ represents the system state after a specific gate is applied.

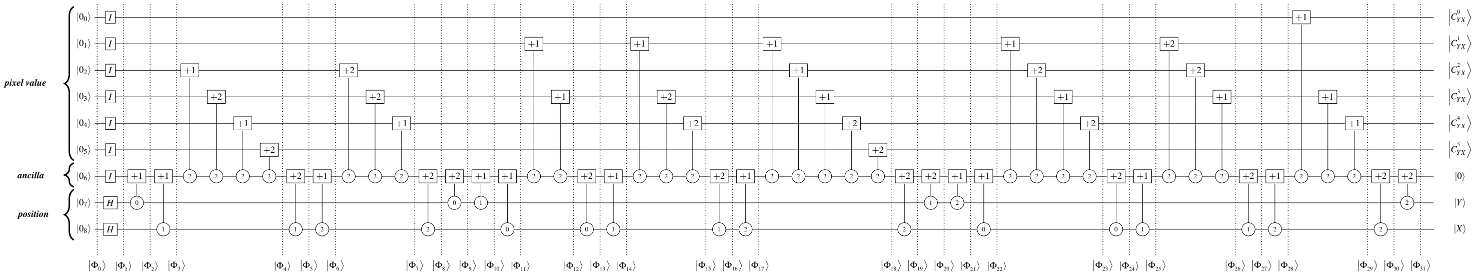

Figure S1. The ternary quantum circuit that represents the 3×3 grayscale image shown in Figure S2 using the proposed NQQR model.

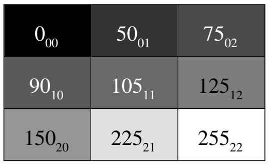

Figure S2. A 3×3 grayscale image.

The image in Figure S2 is represented according to the **G-Steps** that are used to represent $3^n \times 3^n$ grayscale images using the NQQR model. The representation starting with **G-Step I** by initializing the quantum image by $2n+q+1$ qutrits all initialized to $|0\rangle$, then $H^{\otimes 2n}$ gate are applied on the $2n$ position qutrits.
For the current image, the quantum image is initialized by $|\Phi_0\rangle$ state with 9 qutrits ($q = 6$ and $n = 1$) all initialized to $|0\rangle$,

$$\left|\Phi_0\right\rangle = |0\rangle^{\otimes q} \otimes |0\rangle_{aux} \otimes |0\rangle^{\otimes 2} = |000000\rangle \otimes |0\rangle_{aux} \otimes |00\rangle .$$

G-Step I, two Hadamard ($H^{\otimes 2}$) gates are applied to the 2 position qutrits to encode all pixel coordinates: $|00\rangle$, $|01\rangle$, $|02\rangle$, $|10\rangle$, $|11\rangle$, $|12\rangle$, $|20\rangle$, $|21\rangle$, $|22\rangle$,

$$|\Phi_1\rangle = \frac{1}{3}\sum_{Y=0}^{2}\sum_{X=0}^{2} |000000\rangle\, |0\rangle_{aux}\, |YX\rangle .$$

G-Step II: A generalized N-qutrit gate with n control qutrits (encoding the row position Y) and a $[+1]$ target gate is used to update the ancilla qutrit from $|0\rangle$ to $|1\rangle$ (Enable row Y). This gate activates only the pixels belonging to the enabled row, while all other rows remain unaffected.
For the current image, row $Y = 0$ is enabled updating the ancilla (aux) qutrit state from $|0\rangle$ to $|1\rangle$,

$$|\Phi_2\rangle = \frac{1}{3}|000000\rangle\, |1\rangle_{aux}\, |0X\rangle + \frac{1}{3}\sum_{v}\sum_{u} |000000\rangle\, |1\rangle_{aux}\, |vu\rangle ,$$

where $|YX\rangle$ denoting the pixel currently being processed and $|vu\rangle$ representing the remaining pixels that are not updated at this step.

G-Step III: Another generalized N-qutrit gate with n control qutrits (column position X) and a $[+1]$ target gate is used to update the ancilla qutrit from $|1\rangle$ to $|2\rangle$ (Enable column X).
For the current image, the grayscale value of pixel $(0,0)$ is zero and therefore requires no encoding operations in the quantum circuit. Consequently, the column $X = 1$ is enabled, fully selecting pixel $(0,1)$ updating the ancilla qutrit from $|1\rangle$ to $|2\rangle$,

$$|\Phi_3\rangle = \frac{1}{3}|000000\rangle|2\rangle_{aux}|01\rangle + \frac{1}{3}\sum_v\sum_u|000000\rangle|2\rangle_{aux}|vu\rangle.$$

G-Step IV: The grayscale value of the pixel (Y,X), in range $[0-255]$, is assigned using up to 5 two-qutrit gates, where each gate uses the ancilla qutrit in state $|2\rangle$ as the control qutrit and applies a $[+1]$ or $[+2]$ target gate to one of the q qutrits.
For the current image, assign the grayscale value for pixel $(0,1)$ to $50 = 001212$ using 4 two-qutrit gates,

$$|\Phi_4\rangle = \frac{1}{3}|001212\rangle|2\rangle_{aux}|01\rangle + \frac{1}{3}\sum_v\sum_u|000000\rangle|2\rangle_{aux}|vu\rangle.$$

G-Step V: A generalized N-qutrit gate with n control qutrits (column position X) and a $[+2]$ target gate is used to reverse the effect of G-Step III, returning the ancilla state from $|2\rangle$ to $|1\rangle$, preparing the representation of the next pixel in the same row Y (Disable column X).
For the current image, the column $X = 1$ is disabled updating the ancilla qutrit from $|2\rangle$ to $|1\rangle$,

$$|\Phi_5\rangle = \frac{1}{3}|000000\rangle|1\rangle_{aux}|0X\rangle + \frac{1}{3}|001212\rangle|1\rangle_{aux}|01\rangle + \frac{1}{3}\sum_v\sum_u|000000\rangle|1\rangle_{aux}|vu\rangle.$$

G-Step III, the column $X = 2$ is enabled updating the ancilla qutrit from $|1\rangle$ to $|2\rangle$,

$$|\Phi_6\rangle = \frac{1}{3}|000000\rangle|2\rangle_{aux}|02\rangle + \frac{1}{3}|001212\rangle|2\rangle_{aux}|01\rangle + \frac{1}{3}\sum_v\sum_u|000000\rangle|2\rangle_{aux}|vu\rangle.$$

G-Step IV, assign the grayscale value for pixel $(0,2)$ to $75 = 002210$,

$$|\Phi_7\rangle = \frac{1}{3}|002210\rangle|2\rangle_{aux}|02\rangle + \frac{1}{3}|001212\rangle|2\rangle_{aux}|01\rangle + \frac{1}{3}\sum_v\sum_u|000000\rangle|2\rangle_{aux}|vu\rangle.$$

G-Step V, the column $X = 2$ is disabled updating the ancilla qutrit from $|2\rangle$ to $|1\rangle$,

$$|\Phi_8\rangle = \frac{1}{3}|000000\rangle|1\rangle_{aux}|0X\rangle + \frac{1}{3}|002210\rangle|1\rangle_{aux}|02\rangle + \frac{1}{3}|001212\rangle|1\rangle_{aux}|01\rangle + \frac{1}{3}\sum_v\sum_u|000000\rangle|1\rangle_{aux}|vu\rangle.$$

G-Step VI: A generalized N-qutrit gate with n control qutrits (row position Y) and a $[+2]$ target gate is used to reverse the effect of Step II, returning the ancilla state from $|1\rangle$ to $|0\rangle$, preparing the representation of the next row of pixels in the image (Disable row Y).
For the current image, after completing Row_0, the row $Y = 0$ is disabled updating the ancilla qutrit from $|1\rangle$ to $|0\rangle$,

$$|\Phi_9\rangle = \frac{1}{3}|000000\rangle|0\rangle_{aux}|YX\rangle + \frac{1}{3}|002210\rangle|0\rangle_{aux}|02\rangle + \frac{1}{3}|001212\rangle|0\rangle_{aux}|01\rangle + \frac{1}{3}\sum_v\sum_u|000000\rangle|0\rangle_{aux}|vu\rangle.$$

G-Step II, the row $Y = 1$ is enabled updating the ancilla qutrit from $|0\rangle$ to $|1\rangle$,

$$|\Phi_{10}\rangle = \frac{1}{3}|000000\rangle|1\rangle_{aux}|1X\rangle + \frac{1}{3}|002210\rangle|1\rangle_{aux}|02\rangle + \frac{1}{3}|001212\rangle|1\rangle_{aux}|01\rangle + \frac{1}{3}\sum_v\sum_u|000000\rangle|1\rangle_{aux}|vu\rangle.$$

G-Step III, the column $X = 0$ is enabled updating the ancilla qutrit from $|1\rangle$ to $|2\rangle$,

$$|\Phi_{11}\rangle = \frac{1}{3}|000000\rangle|2\rangle_{aux}|10\rangle + \frac{1}{3}|002210\rangle|2\rangle_{aux}|02\rangle + \frac{1}{3}|001212\rangle|2\rangle_{aux}|01\rangle + \frac{1}{3}\sum_v\sum_u|000000\rangle|2\rangle_{aux}|vu\rangle.$$

G-Step IV, assign the grayscale value for pixel $(1,0)$ to $90 = 010100$,

$$|\Phi_{12}\rangle = \frac{1}{3}|010100\rangle|2\rangle_{aux}|10\rangle + \frac{1}{3}|002210\rangle|2\rangle_{aux}|02\rangle + \frac{1}{3}|001212\rangle|2\rangle_{aux}|01\rangle + \frac{1}{3}\sum_v\sum_u|000000\rangle|2\rangle_{aux}|vu\rangle.$$

G-Step V, the column $X = 0$ is disabled updating the ancilla qutrit from $|2\rangle$ to $|1\rangle$,

$$|\Phi_{13}\rangle = \frac{1}{3}|000000\rangle|1\rangle_{aux}|1X\rangle + \frac{1}{3}|010100\rangle|1\rangle_{aux}|10\rangle + \frac{1}{3}|002210\rangle|1\rangle_{aux}|02\rangle + \frac{1}{3}|001212\rangle|1\rangle_{aux}|01\rangle + \frac{1}{3}\sum_v\sum_u|000000\rangle|1\rangle_{aux}|vu\rangle.$$

G-Step III, the column $X = 1$ is enabled updating the ancilla qutrit from $|1\rangle$ to $|2\rangle$,

$$|\Phi_{14}\rangle = \frac{1}{3}|000000\rangle|2\rangle_{aux}|11\rangle + \frac{1}{3}|010100\rangle|2\rangle_{aux}|10\rangle + \frac{1}{3}|002210\rangle|2\rangle_{aux}|02\rangle + \frac{1}{3}|001212\rangle|2\rangle_{aux}|01\rangle + \frac{1}{3}\sum_v\sum_u|000000\rangle|2\rangle_{aux}|vu\rangle.$$

G-Step IV, assign the grayscale value for pixel $(1,1)$ to $105 = 010220$,

$$|\Phi_{15}\rangle = \frac{1}{3}|010220\rangle|2\rangle_{aux}|11\rangle + \frac{1}{3}|010100\rangle|2\rangle_{aux}|10\rangle + \frac{1}{3}|002210\rangle|2\rangle_{aux}|02\rangle + \frac{1}{3}|001212\rangle|2\rangle_{aux}|01\rangle + \frac{1}{3}\sum_v\sum_u|000000\rangle|2\rangle_{aux}|vu\rangle.$$

G-Step V, the column $X = 1$ is disabled updating the ancilla qutrit from $|2\rangle$ to $|1\rangle$,

$$|\Phi_{16}\rangle = \frac{1}{3}|000000\rangle|1\rangle_{aux}|1X\rangle + \frac{1}{3}|010220\rangle|1\rangle_{aux}|11\rangle + \frac{1}{3}|010100\rangle|1\rangle_{aux}|10\rangle + \frac{1}{3}|002210\rangle|1\rangle_{aux}|02\rangle + \frac{1}{3}|001212\rangle|1\rangle_{aux}|01\rangle + \frac{1}{3}\sum_v\sum_u|000000\rangle|1\rangle_{aux}|vu\rangle.$$

G-Step III, the column $X = 2$ is enabled updating the ancilla qutrit from $|1\rangle$ to $|2\rangle$,

$$|\Phi_{17}\rangle = \frac{1}{3}|000000\rangle|2\rangle_{aux}|12\rangle + \frac{1}{3}|010220\rangle|2\rangle_{aux}|11\rangle + \frac{1}{3}|010100\rangle|2\rangle_{aux}|10\rangle + \frac{1}{3}|002210\rangle|2\rangle_{aux}|02\rangle + \frac{1}{3}|001212\rangle|2\rangle_{aux}|01\rangle + \frac{1}{3}\sum_v\sum_u|000000\rangle|2\rangle_{aux}|vu\rangle.$$

G-Step IV, assign the grayscale value of pixel $(1,2)$ to $125 = 011122$,

$$|\Phi_{18}\rangle = \frac{1}{3}|011122\rangle|2\rangle_{aux}|12\rangle + \frac{1}{3}|010220\rangle|2\rangle_{aux}|11\rangle + \frac{1}{3}|010100\rangle|2\rangle_{aux}|10\rangle + \frac{1}{3}|002210\rangle|2\rangle_{aux}|02\rangle + \frac{1}{3}|001212\rangle|2\rangle_{aux}|01\rangle + \frac{1}{3}\sum_v\sum_u|000000\rangle|2\rangle_{aux}|vu\rangle.$$

G-Step V, the column $X = 2$ is disabled updating the ancilla qutrit from $|2\rangle$ to $|1\rangle$,

$$|\Phi_{19}\rangle = \frac{1}{3}|000000\rangle|1\rangle_{aux}|1X\rangle + \frac{1}{3}|011122\rangle|1\rangle_{aux}|12\rangle + \frac{1}{3}|010220\rangle|1\rangle_{aux}|11\rangle + \frac{1}{3}|010100\rangle|1\rangle_{aux}|10\rangle + \frac{1}{3}|002210\rangle|1\rangle_{aux}|02\rangle + \frac{1}{3}|001212\rangle|1\rangle_{aux}|01\rangle + \frac{1}{3}\sum_v\sum_u|000000\rangle|1\rangle_{aux}|vu\rangle.$$

G-Step VI, After completing Row_1, the row $Y=1$ is disabled updating the ancilla qutrit from $|1\rangle$ to $|0\rangle$,

$$
\begin{aligned}
|\Phi_{20}\rangle = &\frac{1}{3}|000000\rangle|0\rangle_{aux}|YX\rangle + \frac{1}{3}|011122\rangle|0\rangle_{aux}|12\rangle + \frac{1}{3}|010220\rangle|0\rangle_{aux}|11\rangle \\
&+ \frac{1}{3}|010100\rangle|0\rangle_{aux}|10\rangle + \frac{1}{3}|002210\rangle|0\rangle_{aux}|02\rangle + \frac{1}{3}|001212\rangle|0\rangle_{aux}|01\rangle \\
&+ \frac{1}{3}\sum_v\sum_u|000000\rangle|0\rangle_{aux}|vu\rangle .
\end{aligned}
$$

G-Step II, the row $Y=2$ is enabled updating the ancilla qutrit from $|0\rangle$ to $|1\rangle$,

$$
\begin{aligned}
|\Phi_{21}\rangle = &\frac{1}{3}|000000\rangle|1\rangle_{aux}|2X\rangle + \frac{1}{3}|011122\rangle|1\rangle_{aux}|12\rangle + \frac{1}{3}|010220\rangle|1\rangle_{aux}|11\rangle \\
&+ \frac{1}{3}|010100\rangle|1\rangle_{aux}|10\rangle + \frac{1}{3}|002210\rangle|1\rangle_{aux}|02\rangle + \frac{1}{3}|001212\rangle|1\rangle_{aux}|01\rangle \\
&+ \frac{1}{3}\sum_v\sum_u|000000\rangle|1\rangle_{aux}|vu\rangle .
\end{aligned}
$$

G-Step III, the column $X=0$ is enabled updating the ancilla qutrit from $|1\rangle$ to $|2\rangle$,

$$
\begin{aligned}
|\Phi_{22}\rangle = &\frac{1}{3}|000000\rangle|2\rangle_{aux}|20\rangle + \frac{1}{3}|011122\rangle|2\rangle_{aux}|12\rangle + \frac{1}{3}|010220\rangle|2\rangle_{aux}|11\rangle \\
&+ \frac{1}{3}|010100\rangle|2\rangle_{aux}|10\rangle + \frac{1}{3}|002210\rangle|2\rangle_{aux}|02\rangle + \frac{1}{3}|001212\rangle|2\rangle_{aux}|01\rangle \\
&+ \frac{1}{3}\sum_v\sum_u|000000\rangle|2\rangle_{aux}|vu\rangle .
\end{aligned}
$$

G-Step IV, assign the grayscale value for pixel $(2,0)$ to $150 = 012120$,

$$
\begin{aligned}
|\Phi_{23}\rangle = &\frac{1}{3}|012120\rangle|2\rangle_{aux}|20\rangle + \frac{1}{3}|011122\rangle|2\rangle_{aux}|12\rangle + \frac{1}{3}|010220\rangle|2\rangle_{aux}|11\rangle \\
&+ \frac{1}{3}|010100\rangle|2\rangle_{aux}|10\rangle + \frac{1}{3}|002210\rangle|2\rangle_{aux}|02\rangle + \frac{1}{3}|001212\rangle|2\rangle_{aux}|01\rangle \\
&+ \frac{1}{3}\sum_v\sum_u|000000\rangle|2\rangle_{aux}|vu\rangle .
\end{aligned}
$$

G-Step V, the column $X=0$ is disabled updating the ancilla qutrit from $|2\rangle$ to $|1\rangle$,

$$
\begin{aligned}
|\Phi_{24}\rangle = &\frac{1}{3}|000000\rangle|1\rangle_{aux}|2X\rangle + \frac{1}{3}|012120\rangle|1\rangle_{aux}|20\rangle + \frac{1}{3}|011122\rangle|1\rangle_{aux}|12\rangle \\
&+ \frac{1}{3}|010220\rangle|1\rangle_{aux}|11\rangle + \frac{1}{3}|010100\rangle|1\rangle_{aux}|10\rangle + \frac{1}{3}|002210\rangle|1\rangle_{aux}|02\rangle \\
&+ \frac{1}{3}|001212\rangle|1\rangle_{aux}|01\rangle + \frac{1}{3}\sum_v\sum_u|000000\rangle|1\rangle_{aux}|vu\rangle .
\end{aligned}
$$

G-Step III, the column $X=1$ is enabled updating the ancilla qutrit from $|1\rangle$ to $|2\rangle$,

$$
\begin{aligned}
|\Phi_{25}\rangle = &\frac{1}{3}|000000\rangle|2\rangle_{aux}|21\rangle + \frac{1}{3}|012120\rangle|2\rangle_{aux}|20\rangle + \frac{1}{3}|011122\rangle|2\rangle_{aux}|12\rangle \\
&+ \frac{1}{3}|010220\rangle|2\rangle_{aux}|11\rangle + \frac{1}{3}|010100\rangle|2\rangle_{aux}|10\rangle + \frac{1}{3}|002210\rangle|2\rangle_{aux}|02\rangle \\
&+ \frac{1}{3}|001212\rangle|2\rangle_{aux}|01\rangle + \frac{1}{3}\sum_v\sum_u|000000\rangle|2\rangle_{aux}|vu\rangle .
\end{aligned}
$$

G-Step IV, assign the grayscale value for pixel $(2,1)$ to $225 = 022100$,

$$
\begin{aligned}
|\Phi_{26}\rangle = &\frac{1}{3}|022100\rangle|2\rangle_{aux}|21\rangle + \frac{1}{3}|012120\rangle|2\rangle_{aux}|20\rangle + \frac{1}{3}|011122\rangle|2\rangle_{aux}|12\rangle \\
&+ \frac{1}{3}|010220\rangle|2\rangle_{aux}|11\rangle + \frac{1}{3}|010100\rangle|2\rangle_{aux}|10\rangle + \frac{1}{3}|002210\rangle|2\rangle_{aux}|02\rangle \\
&+ \frac{1}{3}|001212\rangle|2\rangle_{aux}|01\rangle + \frac{1}{3}\sum_v\sum_u|000000\rangle|2\rangle_{aux}|vu\rangle .
\end{aligned}
$$

G-Step V, the column $X = 1$ is disabled updating the ancilla qutrit from $|2\rangle$ to $|1\rangle$,

$$\begin{aligned}|\Phi_{27}\rangle = &\frac{1}{3}|000000\rangle|1\rangle_{aux}|2X\rangle + \frac{1}{3}|022100\rangle|1\rangle_{aux}|21\rangle + \frac{1}{3}|012120\rangle|1\rangle_{aux}|20\rangle \\ &+ \frac{1}{3}|011122\rangle|1\rangle_{aux}|12\rangle + \frac{1}{3}|010220\rangle|1\rangle_{aux}|11\rangle + \frac{1}{3}|010100\rangle|1\rangle_{aux}|10\rangle \\ &+ \frac{1}{3}|002210\rangle|1\rangle_{aux}|02\rangle + \frac{1}{3}|001212\rangle|1\rangle_{aux}|01\rangle + \frac{1}{3}\sum_v\sum_u|000000\rangle|1\rangle_{aux}|vu\rangle\,.\end{aligned}$$

G-Step III, the column $X = 2$ is enabled updating the ancilla qutrit from $|1\rangle$ to $|2\rangle$,

$$\begin{aligned}|\Phi_{28}\rangle = &\frac{1}{3}|000000\rangle|2\rangle_{aux}|22\rangle + \frac{1}{3}|022100\rangle|2\rangle_{aux}|21\rangle + \frac{1}{3}|012120\rangle|2\rangle_{aux}|20\rangle \\ &+ \frac{1}{3}|011122\rangle|2\rangle_{aux}|12\rangle + \frac{1}{3}|010220\rangle|2\rangle_{aux}|11\rangle + \frac{1}{3}|010100\rangle|2\rangle_{aux}|10\rangle \\ &+ \frac{1}{3}|002210\rangle|2\rangle_{aux}|02\rangle + \frac{1}{3}|001212\rangle|2\rangle_{aux}|01\rangle + \frac{1}{3}\sum_v\sum_u|000000\rangle|2\rangle_{aux}|vu\rangle\,.\end{aligned}$$

G-Step IV, assign the grayscale value for pixel $(2,2)$ to $255 = 100110$,

$$\begin{aligned}|\Phi_{29}\rangle = &\frac{1}{3}|100110\rangle|2\rangle_{aux}|22\rangle + \frac{1}{3}|022100\rangle|2\rangle_{aux}|21\rangle + \frac{1}{3}|012120\rangle|2\rangle_{aux}|20\rangle \\ &+ \frac{1}{3}|011122\rangle|2\rangle_{aux}|12\rangle + \frac{1}{3}|010220\rangle|2\rangle_{aux}|11\rangle + \frac{1}{3}|010100\rangle|2\rangle_{aux}|10\rangle \\ &+ \frac{1}{3}|002210\rangle|2\rangle_{aux}|02\rangle + \frac{1}{3}|001212\rangle|2\rangle_{aux}|01\rangle + \frac{1}{3}\sum_v\sum_u|000000\rangle|2\rangle_{aux}|vu\rangle\,.\end{aligned}$$

G-Step V, the column $X = 2$ is disabled updating the ancilla qutrit from $|2\rangle$ to $|1\rangle$,

$$\begin{aligned}|\Phi_{30}\rangle = &\frac{1}{3}|100110\rangle|1\rangle_{aux}|22\rangle + \frac{1}{3}|022100\rangle|1\rangle_{aux}|21\rangle + \frac{1}{3}|012120\rangle|1\rangle_{aux}|20\rangle \\ &+ \frac{1}{3}|011122\rangle|1\rangle_{aux}|12\rangle + \frac{1}{3}|010220\rangle|1\rangle_{aux}|11\rangle + \frac{1}{3}|010100\rangle|1\rangle_{aux}|10\rangle \\ &+ \frac{1}{3}|002210\rangle|1\rangle_{aux}|02\rangle + \frac{1}{3}|001212\rangle|1\rangle_{aux}|01\rangle + \frac{1}{3}|000000\rangle|1\rangle_{aux}|00\rangle\,.\end{aligned}$$

G-Step VI, After completing Row_2, the row $Y = 2$ is disabled updating the ancilla qutrit from $|1\rangle$ to $|0\rangle$,

$$\begin{aligned}|\Phi_{31}\rangle = &\frac{1}{3}|100110\rangle|0\rangle_{aux}|22\rangle + \frac{1}{3}|022100\rangle|0\rangle_{aux}|21\rangle + \frac{1}{3}|012120\rangle|0\rangle_{aux}|20\rangle \\ &+ \frac{1}{3}|011122\rangle|0\rangle_{aux}|12\rangle + \frac{1}{3}|010220\rangle|0\rangle_{aux}|11\rangle + \frac{1}{3}|010100\rangle|0\rangle_{aux}|10\rangle \\ &+ \frac{1}{3}|002210\rangle|0\rangle_{aux}|02\rangle + \frac{1}{3}|001212\rangle|0\rangle_{aux}|01\rangle + \frac{1}{3}|000000\rangle|0\rangle_{aux}|00\rangle\,.\end{aligned}$$

After completing the encoding of all rows and columns, the final quantum state $|\Phi_{31}\rangle$ represents the full 3×3 grayscale image in Figure S2, with each pixel's grayscale value correctly encoded while preserving the superposition over pixel positions.

S2 State Vector Evolution of NQQR RGB Image

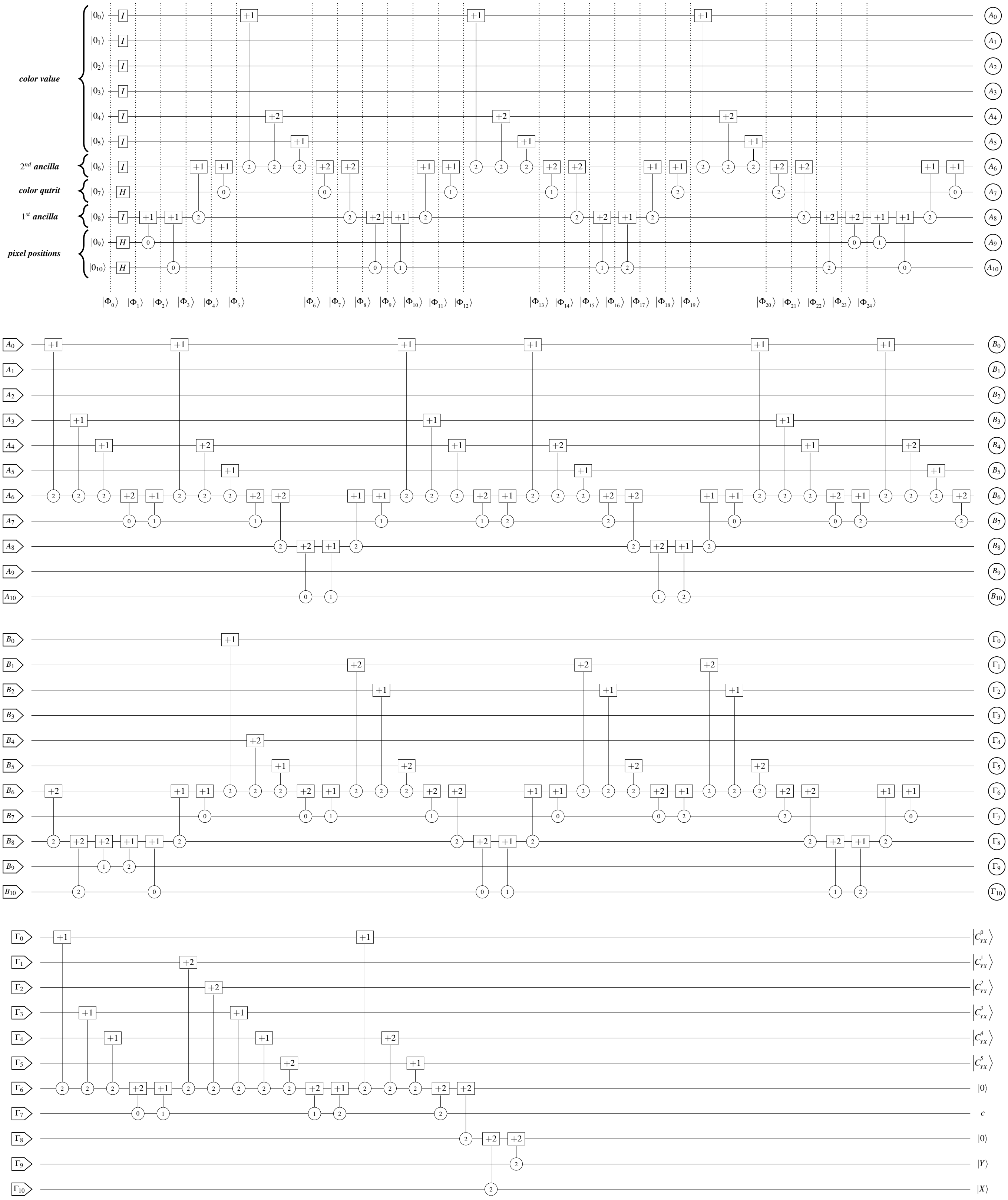

Figure S3. Quantum circuit for the NQQR representation of a 3×3 RGB image shown in Figure S4

$(250,0,0)_{00}$	$(0,250,0)_{01}$	$(0,0,250)_{02}$
$(255,250,0)_{10}$	$(0,255,250)_{11}$	$(255,0,250)_{12}$
$(250,191,0)_{20}$	$(191,0,191)_{21}$	$(255,230,250)_{22}$

Figure S4. A 3×3 RGB image.

This section presents the state vector evolution of the first row of pixels (Row_0) in the quantum image circuit shown in Figure S3, which implements the 3×3 RGB image in Figure S4 using the NQQR model. Each intermediate state $|\Phi_i\rangle$ represents the system state after a specific gate is applied.

The image in Figure S4 is represented according to the **C-Steps** that are used to represent $3^n \times 3^n$ RGB images using the NQQR model. The representation starting with **C-Step I** that initializes the quantum image by $2n+q+3$ qutrits all initialized to $|0\rangle$, then apply $H^{\otimes(2n+1)}$ gates on the $2n$ position qutrits and the color qutrit.
For the current image, the quantum image is initialized by $|\Phi_0\rangle$ state with 11 qutrits ($q = 6$ and $n = 1$) all initialized to $|0\rangle$,

$$|\Phi_0\rangle = |000000\rangle \otimes |0\rangle_{color} \otimes |0\rangle_{aux_2} \otimes |0\rangle_{aux_1} \otimes |0\rangle^{\otimes 2}.$$

Three Hadamard ($H^{\otimes 3}$) gates are applied to the 2 position qutrits and 1 color qutrit to encode all pixel coordinates and the 3 color channels,

$$\begin{aligned}|\Phi_1\rangle &= \frac{1}{3\sqrt{3}} \sum_{Y=0}^{2} \sum_{X=0}^{2} |000000\rangle \otimes (|0\rangle_R + |1\rangle_G + |2\rangle_B) \otimes |0\rangle_{aux_2} \otimes |0\rangle_{aux_1} \otimes |YX\rangle \\ &= \frac{1}{3\sqrt{3}} \sum_{Y=0}^{2} \sum_{X=0}^{2} (|000000\rangle\, |0\rangle_R + |000000\rangle\, |1\rangle_G + |000000\rangle\, |2\rangle_B)\, |0\rangle_{aux_2}\, |0\rangle_{aux_1}\, |YX\rangle .\end{aligned}$$

C-Step II, a generalized N-qutrit gate with n control qutrits (row position Y) and a $[+1]$ target gate is used to update the 1st ancilla qutrit from $|0\rangle$ to $|1\rangle$ (Enable row Y).
For the current image, the row $Y = 0$ is enabled updating the the 1st ancilla (aux_1) from $|0\rangle$ to $|1\rangle$,

$$\begin{aligned}|\Phi_2\rangle &= \frac{1}{3\sqrt{3}} (|000000\rangle\, |0\rangle_R + |000000\rangle\, |1\rangle_G + |000000\rangle\, |2\rangle_B)\, |0\rangle_{aux_2}\, |1\rangle_{aux_1}\, |0X\rangle \\ &+ \frac{1}{3\sqrt{3}} \sum_v \sum_u (|000000\rangle\, |0\rangle_R + |000000\rangle\, |1\rangle_G + |000000\rangle\, |2\rangle_B)\, |0\rangle_{aux_2}\, |1\rangle_{aux_1}\, |vu\rangle ,\end{aligned}$$

where $|YX\rangle$ denoting the pixel that is currently being processed and $|vu\rangle$ representing the remaining pixels that are not updated at this step.

C-Step III, a generalized N-qutrit gate with n control qutrits (column position X) and a $[+1]$ target gate is used to update the 1st ancilla qutrit from $|1\rangle$ to $|2\rangle$ (Enable column X).
For the current image, the column $X = 0$ is enabled updating the 1st ancilla from $|1\rangle$ to $|2\rangle$,

$$\begin{aligned}|\Phi_3\rangle &= \frac{1}{3\sqrt{3}} (|000000\rangle\, |0\rangle_R + |000000\rangle\, |1\rangle_G + |000000\rangle\, |2\rangle_B)\, |0\rangle_{aux_2}\, |2\rangle_{aux_1}\, |00\rangle \\ &+ \frac{1}{3\sqrt{3}} \sum_v \sum_u (|000000\rangle\, |0\rangle_R + |000000\rangle\, |1\rangle_G + |000000\rangle\, |2\rangle_B)\, |0\rangle_{aux_2}\, |2\rangle_{aux_1}\, |vu\rangle .\end{aligned}$$

C-Step IV, a two-qutrit gate, with the 1st ancilla as the control qutrit in state $|2\rangle$, updates the 2nd ancilla qutrit from $|0\rangle$ to $|1\rangle$ using a $[+1]$ target gate. This operation enables the selected pixel (Y,X), enabling the assignment of its RGB channel values (Enable pixel (Y,X)).
For the current image, the pixel $(0,0)$ is enabled updating the 2nd ancilla qutrit (aux_2) from $|0\rangle$ to $|1\rangle$,

$$\begin{aligned}|\Phi_4\rangle &= \frac{1}{3\sqrt{3}} (|000000\rangle\, |0\rangle_R + |000000\rangle\, |1\rangle_G + |000000\rangle\, |2\rangle_B)\, |1\rangle_{aux_2}\, |2\rangle_{aux_1}\, |00\rangle \\ &+ \frac{1}{3\sqrt{3}} \sum_v \sum_u (|000000\rangle\, |0\rangle_R + |000000\rangle\, |1\rangle_G + |000000\rangle\, |2\rangle_B)\, |1\rangle_{aux_2}\, |2\rangle_{aux_1}\, |vu\rangle .\end{aligned}$$

C-Step V, a two-qutrit gate with the color qutrit as the control qutrit in state $|0\rangle$ detects the Red channel and the target $[+1]$ gate updates the 2nd ancilla from $|1\rangle$ to $|2\rangle$. Then, the Red value is assigned using up to 5 two-qutrit gates from the 2nd ancilla to the q qutrits. Finally, another two-qutrit gate with control $|0\rangle$ and target $[+2]$ disables the Red channel.
For the current image, the Red channel is enabled updating the the 2nd ancilla from $|1\rangle$ to $|2\rangle$,

$$\begin{aligned}\left|\Phi_5\right\rangle = &\frac{1}{3\sqrt{3}}(|000000\rangle\,|0\rangle_R + |000000\rangle\,|1\rangle_G + |000000\rangle\,|2\rangle_B)\,|2\rangle_{aux_2}\,|2\rangle_{aux_1}\,|00\rangle \\ &+ \frac{1}{3\sqrt{3}}\sum_v\sum_u(|000000\rangle\,|0\rangle_R + |000000\rangle\,|1\rangle_G + |000000\rangle\,|2\rangle_B)\,|2\rangle_{aux_2}\,|2\rangle_{aux_1}\,|vu\rangle\,.\end{aligned}$$

C-Step V, assign the Red color value to the pixel $(0,0)$ to $250 = 100021$,

$$\begin{aligned}\left|\Phi_6\right\rangle = &\frac{1}{3\sqrt{3}}(|100021\rangle\,|0\rangle_R + |000000\rangle\,|1\rangle_G + |000000\rangle\,|2\rangle_B)\,|2\rangle_{aux_2}\,|2\rangle_{aux_1}\,|00\rangle \\ &+ \frac{1}{3\sqrt{3}}\sum_v\sum_u(|000000\rangle\,|0\rangle_R + |000000\rangle\,|1\rangle_G + |000000\rangle\,|2\rangle_B)\,|2\rangle_{aux_2}\,|2\rangle_{aux_1}\,|vu\rangle\,.\end{aligned}$$

C-Step V, the Red channel is disabled updating the 2nd ancilla qutrit from $|2\rangle$ to $|1\rangle$,

$$\begin{aligned}\left|\Phi_7\right\rangle = &\frac{1}{3\sqrt{3}}(|100021\rangle\,|0\rangle_R + |000000\rangle\,|1\rangle_G + |000000\rangle\,|2\rangle_B)\,|1\rangle_{aux_2}\,|2\rangle_{aux_1}\,|00\rangle \\ &+ \frac{1}{3\sqrt{3}}\sum_v\sum_u(|000000\rangle\,|0\rangle_R + |000000\rangle\,|1\rangle_G + |000000\rangle\,|2\rangle_B)\,|1\rangle_{aux_2}\,|2\rangle_{aux_1}\,|vu\rangle\,.\end{aligned}$$

C-Step V-III, a two-qutrit gate with the control qutrit (1st ancilla) is in state $|2\rangle$ reverses the effect of C-Step IV, returning the 2nd ancilla from $|1\rangle$ to $|0\rangle$ using a $[+2]$ target gate (Disable pixel).
For the current image, the pixel $(0,0)$ is disabled updating the 2nd ancilla from $|1\rangle$ to $|0\rangle$,

$$\begin{aligned}\left|\Phi_8\right\rangle = &\frac{1}{3\sqrt{3}}(|100021\rangle\,|0\rangle_R + |000000\rangle\,|1\rangle_G + |000000\rangle\,|2\rangle_B)\,|0\rangle_{aux_2}\,|2\rangle_{aux_1}\,|00\rangle \\ &+ \frac{1}{3\sqrt{3}}\sum_v\sum_u(|000000\rangle\,|0\rangle_R + |000000\rangle\,|1\rangle_G + |000000\rangle\,|2\rangle_B)\,|0\rangle_{aux_2}\,|2\rangle_{aux_1}\,|vu\rangle\,.\end{aligned}$$

C-Step IX, a generalized N-qutrit gate with n control qutrits (column position X) and a $[+2]$ target gate is used to reverse the effect of C-Step III, returning the 1st ancilla from $|2\rangle$ to $|1\rangle$, preparing the representation for the next pixel in the same row Y (Disable column X).
For the current image, the column $X = 0$ is disabled updating the 1st ancilla from $|2\rangle$ to $|1\rangle$,

$$\begin{aligned}\left|\Phi_9\right\rangle = &\frac{1}{3\sqrt{3}}(|000000\rangle\,|0\rangle_R + |000000\rangle\,|1\rangle_G + |000000\rangle\,|2\rangle_B)\,|0\rangle_{aux_2}\,|1\rangle_{aux_1}\,|0X\rangle \\ &+ \frac{1}{3\sqrt{3}}(|100021\rangle\,|0\rangle_R + |000000\rangle\,|1\rangle_G + |000000\rangle\,|2\rangle_B)\,|0\rangle_{aux_2}\,|1\rangle_{aux_1}\,|00\rangle \\ &+ \frac{1}{3\sqrt{3}}\sum_v\sum_u(|000000\rangle\,|0\rangle_R + |000000\rangle\,|1\rangle_G + |000000\rangle\,|2\rangle_B)\,|0\rangle_{aux_2}\,|1\rangle_{aux_1}\,|vu\rangle\,.\end{aligned}$$

C-Step III, the column $X = 1$ is enabled updating the 1st ancilla from $|1\rangle$ to $|2\rangle$,

$$\begin{aligned}\left|\Phi_{10}\right\rangle = &\frac{1}{3\sqrt{3}}(|000000\rangle\,|0\rangle_R + |000000\rangle\,|1\rangle_G + |000000\rangle\,|2\rangle_B)\,|0\rangle_{aux_2}\,|2\rangle_{aux_1}\,|01\rangle \\ &+ \frac{1}{3\sqrt{3}}(|100021\rangle\,|0\rangle_R + |000000\rangle\,|1\rangle_G + |000000\rangle\,|2\rangle_B)\,|0\rangle_{aux_2}\,|2\rangle_{aux_1}\,|00\rangle \\ &+ \frac{1}{3\sqrt{3}}\sum_v\sum_u(|000000\rangle\,|0\rangle_R + |000000\rangle\,|1\rangle_G + |000000\rangle\,|2\rangle_B)\,|0\rangle_{aux_2}\,|2\rangle_{aux_1}\,|vu\rangle\,.\end{aligned}$$

C-Step IV, the pixel position $(0,1)$ is enabled updating the 2nd ancilla qutrit from $|0\rangle$ to $|1\rangle$,

$$\begin{aligned}|\Phi_{11}\rangle = &\frac{1}{3\sqrt{3}}(|000000\rangle|0\rangle_R + |000000\rangle|1\rangle_G + |000000\rangle|2\rangle_B)|1\rangle_{aux_2}|2\rangle_{aux_1}|01\rangle \\ &+\frac{1}{3\sqrt{3}}(|100021\rangle|0\rangle_R + |000000\rangle|1\rangle_G + |000000\rangle|2\rangle_B)|1\rangle_{aux_2}|2\rangle_{aux_1}|00\rangle \\ &+\frac{1}{3\sqrt{3}}\sum_v\sum_u(|000000\rangle|0\rangle_R + |000000\rangle|1\rangle_G + |000000\rangle|2\rangle_B)|1\rangle_{aux_2}|2\rangle_{aux_1}|vu\rangle.\end{aligned}$$

C-Step VI, the Green channel is enabled updating the 2nd ancilla qutrit from $|1\rangle$ to $|2\rangle$,

$$\begin{aligned}|\Phi_{12}\rangle = &\frac{1}{3\sqrt{3}}(|000000\rangle|0\rangle_R + |000000\rangle|1\rangle_G + |000000\rangle|2\rangle_B)|2\rangle_{aux_2}|2\rangle_{aux_1}|01\rangle \\ &+\frac{1}{3\sqrt{3}}(|100021\rangle|0\rangle_R + |000000\rangle|1\rangle_G + |000000\rangle|2\rangle_B)|2\rangle_{aux_2}|2\rangle_{aux_1}|00\rangle \\ &+\frac{1}{3\sqrt{3}}\sum_v\sum_u(|000000\rangle|0\rangle_R + |000000\rangle|1\rangle_G + |000000\rangle|2\rangle_B)|2\rangle_{aux_2}|2\rangle_{aux_1}|vu\rangle.\end{aligned}$$

C-Step VI, assign the Green value for pixel $(0,1)$ to $250 = 100021$,

$$\begin{aligned}|\Phi_{13}\rangle = &\frac{1}{3\sqrt{3}}(|000000\rangle|0\rangle_R + |100021\rangle|1\rangle_G + |000000\rangle|2\rangle_B)|2\rangle_{aux_2}|2\rangle_{aux_1}|01\rangle \\ &+\frac{1}{3\sqrt{3}}(|100021\rangle|0\rangle_R + |000000\rangle|1\rangle_G + |000000\rangle|2\rangle_B)|2\rangle_{aux_2}|2\rangle_{aux_1}|00\rangle \\ &+\frac{1}{3\sqrt{3}}\sum_v\sum_u(|000000\rangle|0\rangle_R + |000000\rangle|1\rangle_G + |000000\rangle|2\rangle_B)|2\rangle_{aux_2}|2\rangle_{aux_1}|vu\rangle.\end{aligned}$$

C-Step VI, the Green channel is disabled updating the 2nd ancilla qutrit from $|2\rangle$ to $|1\rangle$,

$$\begin{aligned}|\Phi_{14}\rangle = &\frac{1}{3\sqrt{3}}(|000000\rangle|0\rangle_R + |100021\rangle|1\rangle_G + |000000\rangle|2\rangle_B)|1\rangle_{aux_2}|2\rangle_{aux_1}|01\rangle \\ &+\frac{1}{3\sqrt{3}}(|100021\rangle|0\rangle_R + |000000\rangle|1\rangle_G + |000000\rangle|2\rangle_B)|1\rangle_{aux_2}|2\rangle_{aux_1}|00\rangle \\ &+\frac{1}{3\sqrt{3}}\sum_v\sum_u(|000000\rangle|0\rangle_R + |000000\rangle|1\rangle_G + |000000\rangle|2\rangle_B)|1\rangle_{aux_2}|2\rangle_{aux_1}|vu\rangle.\end{aligned}$$

C-Step VIII, the pixel $(0,1)$ is disabled updating the 2nd ancilla qutrit from $|1\rangle$ to $|0\rangle$,

$$\begin{aligned}|\Phi_{15}\rangle = &\frac{1}{3\sqrt{3}}(|000000\rangle|0\rangle_R + |100021\rangle|1\rangle_G + |000000\rangle|2\rangle_B)|0\rangle_{aux_2}|2\rangle_{aux_1}|01\rangle \\ &+\frac{1}{3\sqrt{3}}(|100021\rangle|0\rangle_R + |000000\rangle|1\rangle_G + |000000\rangle|2\rangle_B)|0\rangle_{aux_2}|2\rangle_{aux_1}|00\rangle \\ &+\frac{1}{3\sqrt{3}}\sum_v\sum_u(|000000\rangle|0\rangle_R + |000000\rangle|1\rangle_G + |000000\rangle|2\rangle_B)|0\rangle_{aux_2}|2\rangle_{aux_1}|vu\rangle.\end{aligned}$$

C-Step IX, the column $X = 1$ is disabled updating the 1st ancilla from $|2\rangle$ to $|1\rangle$,

$$\begin{aligned}|\Phi_{16}\rangle = &\frac{1}{3\sqrt{3}}(|000000\rangle|0\rangle_R + |000000\rangle|1\rangle_G + |000000\rangle|2\rangle_B)|0\rangle_{aux_2}|1\rangle_{aux_1}|0X\rangle \\ &+\frac{1}{3\sqrt{3}}(|000000\rangle|0\rangle_R + |100021\rangle|1\rangle_G + |000000\rangle|2\rangle_B)|0\rangle_{aux_2}|1\rangle_{aux_1}|01\rangle \\ &+\frac{1}{3\sqrt{3}}(|100021\rangle|0\rangle_R + |000000\rangle|1\rangle_G + |000000\rangle|2\rangle_B)|0\rangle_{aux_2}|1\rangle_{aux_1}|00\rangle \\ &+\frac{1}{3\sqrt{3}}\sum_v\sum_u(|000000\rangle|0\rangle_R + |000000\rangle|1\rangle_G + |000000\rangle|2\rangle_B)|0\rangle_{aux_2}|1\rangle_{aux_1}|vu\rangle.\end{aligned}$$

C-Step III, the column $X = 2$ is enabled updating the 1st ancilla from $|1\rangle$ to $|2\rangle$,

$$
\begin{aligned}
\left|\Phi_{17}\right\rangle = &\frac{1}{3\sqrt{3}}(|000000\rangle\,|0\rangle_R + |000000\rangle\,|1\rangle_G + |000000\rangle\,|2\rangle_B)\,|0\rangle_{aux_2}\,|2\rangle_{aux_1}\,|02\rangle \\
&+ \frac{1}{3\sqrt{3}}(|000000\rangle\,|0\rangle_R + |100021\rangle\,|1\rangle_G + |000000\rangle\,|2\rangle_B)\,|0\rangle_{aux_2}\,|2\rangle_{aux_1}\,|01\rangle \\
&+ \frac{1}{3\sqrt{3}}(|100021\rangle\,|0\rangle_R + |000000\rangle\,|1\rangle_G + |000000\rangle\,|2\rangle_B)\,|0\rangle_{aux_2}\,|2\rangle_{aux_1}\,|00\rangle \\
&+ \frac{1}{3\sqrt{3}}\sum_v\sum_u(|000000\rangle\,|0\rangle_R + |000000\rangle\,|1\rangle_G + |000000\rangle\,|2\rangle_B)\,|0\rangle_{aux_2}\,|2\rangle_{aux_1}\,|vu\rangle\,.
\end{aligned}
$$

C-Step IV, the pixel position $(0,2)$ is enabled updating the 2nd ancilla qutrit from $|0\rangle$ to $|1\rangle$,

$$
\begin{aligned}
\left|\Phi_{18}\right\rangle = &\frac{1}{3\sqrt{3}}(|000000\rangle\,|0\rangle_R + |000000\rangle\,|1\rangle_G + |000000\rangle\,|2\rangle_B)\,|1\rangle_{aux_2}\,|2\rangle_{aux_1}\,|02\rangle \\
&+ \frac{1}{3\sqrt{3}}(|000000\rangle\,|0\rangle_R + |100021\rangle\,|1\rangle_G + |000000\rangle\,|2\rangle_B)\,|1\rangle_{aux_2}\,|2\rangle_{aux_1}\,|01\rangle \\
&+ \frac{1}{3\sqrt{3}}(|100021\rangle\,|0\rangle_R + |000000\rangle\,|1\rangle_G + |000000\rangle\,|2\rangle_B)\,|1\rangle_{aux_2}\,|2\rangle_{aux_1}\,|00\rangle \\
&+ \frac{1}{3\sqrt{3}}\sum_v\sum_u(|000000\rangle\,|0\rangle_R + |000000\rangle\,|1\rangle_G + |000000\rangle\,|2\rangle_B)\,|1\rangle_{aux_2}\,|2\rangle_{aux_1}\,|vu\rangle\,.
\end{aligned}
$$

C-Step VII, the Blue channel is enabled updating the 2nd ancilla qutrit from $|1\rangle$ to $|2\rangle$,

$$
\begin{aligned}
\left|\Phi_{19}\right\rangle = &\frac{1}{3\sqrt{3}}(|000000\rangle\,|0\rangle_R + |000000\rangle\,|1\rangle_G + |000000\rangle\,|2\rangle_B)\,|2\rangle_{aux_2}\,|2\rangle_{aux_1}\,|02\rangle \\
&+ \frac{1}{3\sqrt{3}}(|000000\rangle\,|0\rangle_R + |100021\rangle\,|1\rangle_G + |000000\rangle\,|2\rangle_B)\,|2\rangle_{aux_2}\,|2\rangle_{aux_1}\,|01\rangle \\
&+ \frac{1}{3\sqrt{3}}(|100021\rangle\,|0\rangle_R + |000000\rangle\,|1\rangle_G + |000000\rangle\,|2\rangle_B)\,|2\rangle_{aux_2}\,|2\rangle_{aux_1}\,|00\rangle \\
&+ \frac{1}{3\sqrt{3}}\sum_v\sum_u(|000000\rangle\,|0\rangle_R + |000000\rangle\,|1\rangle_G + |000000\rangle\,|2\rangle_B)\,|2\rangle_{aux_2}\,|2\rangle_{aux_1}\,|vu\rangle\,.
\end{aligned}
$$

C-Step VII, assign the Blue value to pixel $(0,2)$ to $250 = 100021$,

$$
\begin{aligned}
\left|\Phi_{20}\right\rangle = &\frac{1}{3\sqrt{3}}(|000000\rangle\,|0\rangle_R + |000000\rangle\,|1\rangle_G + |100021\rangle\,|2\rangle_B)\,|2\rangle_{aux_2}\,|2\rangle_{aux_1}\,|02\rangle \\
&+ \frac{1}{3\sqrt{3}}(|000000\rangle\,|0\rangle_R + |100021\rangle\,|1\rangle_G + |000000\rangle\,|2\rangle_B)\,|2\rangle_{aux_2}\,|2\rangle_{aux_1}\,|01\rangle \\
&+ \frac{1}{3\sqrt{3}}(|100021\rangle\,|0\rangle_R + |000000\rangle\,|1\rangle_G + |000000\rangle\,|2\rangle_B)\,|2\rangle_{aux_2}\,|2\rangle_{aux_1}\,|00\rangle \\
&+ \frac{1}{3\sqrt{3}}\sum_v\sum_u(|000000\rangle\,|0\rangle_R + |000000\rangle\,|1\rangle_G + |000000\rangle\,|2\rangle_B)\,|2\rangle_{aux_2}\,|2\rangle_{aux_1}\,|vu\rangle\,.
\end{aligned}
$$

C-Step VII, the Blue channel is disabled updating the 2nd ancilla qutrit from $|2\rangle$ to $|1\rangle$,

$$
\begin{aligned}
\left|\Phi_{21}\right\rangle = &\frac{1}{3\sqrt{3}}(|000000\rangle\,|0\rangle_R + |000000\rangle\,|1\rangle_G + |100021\rangle\,|2\rangle_B)\,|1\rangle_{aux_2}\,|2\rangle_{aux_1}\,|02\rangle \\
&+ \frac{1}{3\sqrt{3}}(|000000\rangle\,|0\rangle_R + |100021\rangle\,|1\rangle_G + |000000\rangle\,|2\rangle_B)\,|1\rangle_{aux_2}\,|2\rangle_{aux_1}\,|01\rangle \\
&+ \frac{1}{3\sqrt{3}}(|100021\rangle\,|0\rangle_R + |000000\rangle\,|1\rangle_G + |000000\rangle\,|2\rangle_B)\,|1\rangle_{aux_2}\,|2\rangle_{aux_1}\,|00\rangle \\
&+ \frac{1}{3\sqrt{3}}\sum_v\sum_u(|000000\rangle\,|0\rangle_R + |000000\rangle\,|1\rangle_G + |000000\rangle\,|2\rangle_B)\,|1\rangle_{aux_2}\,|2\rangle_{aux_1}\,|vu\rangle\,.
\end{aligned}
$$

C-Step VIII, the pixel $(0,2)$ is disabled updating the 2nd ancilla qutrit from $|1\rangle$ to $|0\rangle$,

$$
\begin{aligned}
|\Phi_{22}\rangle = &\frac{1}{3\sqrt{3}}(|000000\rangle|0\rangle_R + |000000\rangle|1\rangle_G + |100021\rangle|2\rangle_B)|0\rangle_{aux_2}|2\rangle_{aux_1}|02\rangle \\
&+ \frac{1}{3\sqrt{3}}(|000000\rangle|0\rangle_R + |100021\rangle|1\rangle_G + |000000\rangle|2\rangle_B)|0\rangle_{aux_2}|2\rangle_{aux_1}|01\rangle \\
&+ \frac{1}{3\sqrt{3}}(|100021\rangle|0\rangle_R + |000000\rangle|1\rangle_G + |000000\rangle|2\rangle_B)|0\rangle_{aux_2}|2\rangle_{aux_1}|00\rangle \\
&+ \frac{1}{3\sqrt{3}}\sum_v\sum_u(|000000\rangle|0\rangle_R + |000000\rangle|1\rangle_G + |000000\rangle|2\rangle_B)|0\rangle_{aux_2}|2\rangle_{aux_1}|vu\rangle .
\end{aligned}
$$

C-Step IX, the column $X = 2$ is disabled updating the 1st ancilla qutrit from $|2\rangle$ to $|1\rangle$,

$$
\begin{aligned}
|\Phi_{23}\rangle = &\frac{1}{3\sqrt{3}}(|000000\rangle|0\rangle_R + |000000\rangle|1\rangle_G + |000000\rangle|2\rangle_B)|0\rangle_{aux_2}|1\rangle_{aux_1}|0X\rangle \\
&+ \frac{1}{3\sqrt{3}}(|000000\rangle|0\rangle_R + |000000\rangle|1\rangle_G + |100021\rangle|2\rangle_B)|0\rangle_{aux_2}|1\rangle_{aux_1}|02\rangle \\
&+ \frac{1}{3\sqrt{3}}(|000000\rangle|0\rangle_R + |100021\rangle|1\rangle_G + |000000\rangle|2\rangle_B)|0\rangle_{aux_2}|1\rangle_{aux_1}|01\rangle \\
&+ \frac{1}{3\sqrt{3}}(|100021\rangle|0\rangle_R + |000000\rangle|1\rangle_G + |000000\rangle|2\rangle_B)|0\rangle_{aux_2}|1\rangle_{aux_1}|00\rangle \\
&+ \frac{1}{3\sqrt{3}}\sum_v\sum_u(|000000\rangle|0\rangle_R + |000000\rangle|1\rangle_G + |000000\rangle|2\rangle_B)|0\rangle_{aux_2}|1\rangle_{aux_1}|vu\rangle .
\end{aligned}
$$

C-Step X, a generalized N-qutrit gate with n control qutrits (row position Y) and a $[+2]$ target gate is used to reverse the effect of C-Step II, returning the 1st ancilla from $|1\rangle$ to $|0\rangle$, preparing the representation for the next row of pixels (Disable row X). **For the current image**, the row $Y = 0$ is disabled updating the 1st ancilla qutrit from $|1\rangle$ to $|0\rangle$,

$$
\begin{aligned}
|\Phi_{24}\rangle = &\frac{1}{3\sqrt{3}}(|000000\rangle|0\rangle_R + |000000\rangle|1\rangle_G + |000000\rangle|2\rangle_B)|0\rangle_{aux_2}|0\rangle_{aux_1}|YX\rangle \\
&+ \frac{1}{3\sqrt{3}}(|000000\rangle|0\rangle_R + |000000\rangle|1\rangle_G + |100021\rangle|2\rangle_B)|0\rangle_{aux_2}|0\rangle_{aux_1}|02\rangle \\
&+ \frac{1}{3\sqrt{3}}(|000000\rangle|0\rangle_R + |100021\rangle|1\rangle_G + |000000\rangle|2\rangle_B)|0\rangle_{aux_2}|0\rangle_{aux_1}|01\rangle \\
&+ \frac{1}{3\sqrt{3}}(|100021\rangle|0\rangle_R + |000000\rangle|1\rangle_G + |000000\rangle|2\rangle_B)|0\rangle_{aux_2}|0\rangle_{aux_1}|00\rangle \\
&+ \frac{1}{3\sqrt{3}}\sum_v\sum_u(|000000\rangle|0\rangle_R + |000000\rangle|1\rangle_G + |000000\rangle|2\rangle_B)|0\rangle_{aux_2}|0\rangle_{aux_1}|vu\rangle .
\end{aligned}
$$

After the encoding of all rows and columns is completed, the final quantum state corresponding to the full 3×3 RGB image is expressed as follows:

$$
\begin{aligned}
|\Phi_{\text{RGB}}\rangle = \frac{1}{3\sqrt{3}}&(|100021\rangle|0\rangle_R + |000000\rangle|1\rangle_G + |000000\rangle|2\rangle_B)|0\rangle_{aux_2}|0\rangle_{aux_1}|00\rangle + \\
&(|000000\rangle|0\rangle_R + |100021\rangle|1\rangle_G + |000000\rangle|2\rangle_B)|0\rangle_{aux_2}|0\rangle_{aux_1}|01\rangle + \\
&(|000000\rangle|0\rangle_R + |000000\rangle|1\rangle_G + |100021\rangle|2\rangle_B)|0\rangle_{aux_2}|0\rangle_{aux_1}|02\rangle + \\
&(|100110\rangle|0\rangle_R + |100021\rangle|1\rangle_G + |000000\rangle|2\rangle_B)|0\rangle_{aux_2}|0\rangle_{aux_1}|10\rangle + \\
&(|000000\rangle|0\rangle_R + |100110\rangle|1\rangle_G + |100021\rangle|2\rangle_B)|0\rangle_{aux_2}|0\rangle_{aux_1}|11\rangle + \\
&(|100110\rangle|0\rangle_R + |000000\rangle|1\rangle_G + |100021\rangle|2\rangle_B)|0\rangle_{aux_2}|0\rangle_{aux_1}|12\rangle + \\
&(|100021\rangle|0\rangle_R + |021002\rangle|1\rangle_G + |000000\rangle|2\rangle_B)|0\rangle_{aux_2}|0\rangle_{aux_1}|20\rangle + \\
&(|021002\rangle|0\rangle_R + |000000\rangle|1\rangle_G + |021002\rangle|2\rangle_B)|0\rangle_{aux_2}|0\rangle_{aux_1}|21\rangle + \\
&(|100110\rangle|0\rangle_R + |022112\rangle|1\rangle_G + |100021\rangle|2\rangle_B)|0\rangle_{aux_2}|0\rangle_{aux_1}|22\rangle .
\end{aligned}
$$

References

1. Nielsen, M. A. & Chuang, I. L. *Quantum Computation and Quantum Information: 10th Anniversary Edition* (Cambridge University Press, 2010).
2. Yanofsky, N. S. & Mannucci, M. A. *Quantum Computing for Computer Scientists* (Cambridge University Press, 2008).
